# Recommendations for determining the validity of consumer wearable and smartphone step count: expert statement and checklist of the INTERLIVE network

**DOI:** 10.1136/bjsports-2020-103147

**Published:** 2020-12-24

**Authors:** William Johnston, Pedro B Judice, Pablo Molina García, Jan M Mühlen, Esben Lykke Skovgaard, Julie Stang, Moritz Schumann, Shulin Cheng, Wilhelm Bloch, Jan Christian Brønd, Ulf Ekelund, Anders Grøntved, Brian Caulfield, Francisco B Ortega, Luis B Sardinha

**Affiliations:** 1 Insight Centre for Data Analytics, University College Dublin, Dublin, Ireland; 2 School of Public Health, Physiotherapy and Sports Science, University College Dublin, Dublin, Ireland; 3 Centro de Investigação em Desporto, Educação Física e Exercício e Saúde, CIDEFES, Universidade Lusófona, Lisbon, Portugal; 4 Exercise and Health Laboratory, CIPER, Faculdade de Motricidade Humana, Universidade de Lisboa, Cruz-Quebrada, Portugal; 5 PROFITH (PROmoting FITness and Health through physical activity) Research Group, Department of Physical Education and Sports, Faculty of Sport Sciences, Sport and Health University Research Institute (iMUDS), University of Granada, Granada, Spain; 6 Institute of Cardiovascular Research and Sports Medicine, Department of Molecular and Cellular Sports Medicine, German Sport University, Cologne, Germany; 7 Department of Sports Science and Clinical Biomechanics, Research Unit for Exercise Epidemiology, Centre of Research in Childhood Health, University of Southern Denmark, Odense M, Denmark; 8 Department of Sport Medicine, Norwegian School of Sport Sciences, Oslo, Norway; 9 Exercise Translational Medicine Centre, the Key Laboratory of Systems Biomedicine, Ministry of Education, and Exercise, Health and Technology Centre, Department of Physical Education, Shanghai Jiao Tong University, Shanghai, China

**Keywords:** accelerometer, physical activity, walking, validity, consensus statement

## Abstract

Consumer wearable and smartphone devices provide an accessible means to objectively measure physical activity (PA) through step counts. With the increasing proliferation of this technology, consumers, practitioners and researchers are interested in leveraging these devices as a means to track and facilitate PA behavioural change. However, while the acceptance of these devices is increasing, the validity of many consumer devices have not been rigorously and transparently evaluated. The Towards Intelligent Health and Well-Being Network of Physical Activity Assessment (INTERLIVE) is a joint European initiative of six universities and one industrial partner. The consortium was founded in 2019 and strives to develop best-practice recommendations for evaluating the validity of consumer wearables and smartphones. This expert statement presents a best-practice consumer wearable and smartphone step counter validation protocol. A two-step process was used to aggregate data and form a scientific foundation for the development of an optimal and feasible validation protocol: (1) a systematic literature review and (2) additional searches of the wider literature pertaining to factors that may introduce bias during the validation of these devices. The systematic literature review process identified 2897 potential articles, with 85 articles deemed eligible for the final dataset. From the synthesised data, we identified a set of six key domains to be considered during design and reporting of validation studies: target population, criterion measure, index measure, validation conditions, data processing and statistical analysis. Based on these six domains, a set of key variables of interest were identified and a ‘basic’ and ‘advanced’ multistage protocol for the validation of consumer wearable and smartphone step counters was developed. The INTERLIVE consortium recommends that the proposed protocol is used when considering the validation of any consumer wearable or smartphone step counter. Checklists have been provided to guide validation protocol development and reporting. The network also provide guidance for future research activities, highlighting the imminent need for the development of feasible alternative ‘gold-standard’ criterion measures for free-living validation. Adherence to these validation and reporting standards will help ensure methodological and reporting consistency, facilitating comparison between consumer devices. Ultimately, this will ensure that as these devices are integrated into standard medical care, consumers, practitioners, industry and researchers can use this technology safely and to its full potential.

## Introduction

Over the last decade, wearable and smartphone devices have become ubiquitous among us, with global wearable device and smartphone sales reaching an estimated 305.2 million and 1.5 billion worldwide in 2019, respectivly.^1 2^ These consumer devices afford individuals the ability to easily and objectively monitor their physical activity (PA) without the need for expensive laboratory grade equipment.^3^ With the increasing proliferation of this technology, sports and exercise medicine practitioners and the general public are interested in leveraging these devices as a means to track and facilitate PA behavioural change.^4^

One of the most common measures of PA levels provided by these consumer devices is step count.^5^ Step count is easily understandable by the general population and there is a growing body of evidence supporting the use of these technological approaches in ‘passive’ PA monitoring and ‘active’ intervention. The 2018 PA Guidelines Advisory Committee Report highlighted that step counting is an accessible means to monitor and set PA goals, and that recent evidence supports an inverse dose–response relationship between daily step count and all-cause mortality, cardiovascular events and type 2 diabetes.^5–9^ Furthermore, ‘active’ interventions using wearable activity trackers as a measurement tool can result in significant increases in PA participation, highlighting their potential utility in personalised medicine, increasing adherence to PA and to embed sustained healthy lifestyle habits.^4 10^ Furthermore, the ‘passive’ and unobtrusive measurement of step count across the globe opens a new era of opportunities in the field of digital phenotyping. This population-level approach has recently been used to quantify regional inequalities in PA^11^ and changes in PA levels due to public health containment measures introduced to combat a global pandemic.^12^

While the increasing acceptance across the health and fitness industry has resulted in a surge in validation studies,^3 13–17^ much of the published research fails to rigorously evaluate validity, and there is a lack of consistency across the published protocols, limiting valid comparisons between devices. A letter to the *British Journal of Sports Medicine* called for ‘*evidence-based marketing claims’*, recommending that devices are thoroughly and transparently validated, to ensure that wearable technology can be used safely and to its full potential.^18^ In an attempt to develop a standardised validation process and certification, the Consumer Technology Association (CTA) developed a set of guidelines for the validation of wearable and/or app-based step counters.^19^ While a step in the right direction, the protocol proposed by the CTA focused solely on controlled treadmill walking and running in healthy individuals, failing to consider ecological validation of the technologies within free-living conditions and in cohorts with diverse gait characteristics. A major consequence of this is that while many of the studies demonstrate the validity of these devices during controlled trials, step counting algorithms typically have a higher margin of error during uncontrolled activities of daily living. For example, when considering the same wrist-worn wearable activity tracker compared with a gold-standard video criterion, a mean absolute percentage error (MAPE) of −0.7%–5.2% was observed during controlled treadmill walking,^20^ while a higher MAPE of 17.5%–22.8% was seen during free-living conditions that are more representative of daily life.^21^

To address the above concerns, the Towards Intelligent Health and Well-Being Network of Physical Activity Assessment (INTERLIVE) was formed to develop a best-practice consumer wearable and smartphone step counter validation protocol. To do so, we conducted a systematic literature review of the methods that have previously been used to validate these consumer devices, identified and synthesised other relevant scientific literature to identify aspects that influence validation and developed best-practice validation recommendations. These recommendations are designed for device manufacturers, scientific institutions, healthcare providers and members of the general public who are interested in the transparent and rigorous validation of these devices.

## Expert statement process

### The INTERLIVE network

INTERLIVE is a joint initiative of the University of Lisbon (Portugal), German Sport University (Germany), University of Southern Denmark (Denmark), Norwegian School of Sport Sciences (Norway), University College Dublin (Ireland), University of Granada (Spain) and Huawei Technologies, Finland. The consortium was founded in 2019 and strives towards developing best-practice methods for evaluating the validity of consumer wearables and smartphones. Moreover, we are aiming to increase awareness of the advantages and limitations of different validation methods and to introduce novel health-related metrics, fostering a widespread use of PA indicators.

### Expert validation protocol development

#### Expert validation process

An initial meeting was held in Cascais, Portugal on 15 November 2019, whereby the INTERLIVE consortium identified the development of best-practice validation protocols for consumer wearable and smartphone devices for step counting (part A) and heart rate monitoring (part B) as a key aim of the group. Within the consortium, it was agreed that the optimal process for developing the best-practice validation protocol should begin with extracting key elements of validation protocols previously used in the scientific literature. This information could then be used as the foundation for discussing the optimal and feasible protocols for conducting validation assessment that describes the accuracy end-users can expect. The consortium then formed two working groups: (1) heart rate monitoring (JMM, ELS, JS, SC, WB, JCB, UE, AG and MS) and (2) step counting (WJ, PBJ, PMG, BC, FBO and LBS). The working groups then developed the systematic literature review search strategies, prior to sharing them with the wider consortium. A second consortium meeting was held virtually on 10 March 2020 to finalise the search strategies, including the selection of the minimum a priori required criterion measure(s). The working groups then conducted the systematic literature search process and a framework was developed for extracting data, focusing on target population, criterion and index devices, testing conditions, data processing and statistical analysis. In parallel, additional searches of the wider literature were conducted to identify studies that highlighted factors that may affect the accuracy of wearable and smartphone based step counting that were outside of the scope of our search strategies. The data extraction process was then completed and multiple working group meetings were held to discuss each aspect of the validation protocols used within the included studies. A set of key domains for the best-practice recommendations were developed based on the synthesised outcomes of the literature review, the a priori knowledge relating to research grade device validation^22–25^ and the evidence-based expert opinion of the INTERLIVE members. The synthesised data were then reviewed with respect to these domains, and expert validation protocols for wearable and smartphone step counters (part A) and wearable heart rate monitors (part B) were iteratively developed and shared with the entire consortium. At a virtual meeting held on 17 June 2020, the revised drafts were discussed and the protocols were aligned to ensure harmonisation of the statements. The revised drafts were then edited for consistency and reviewed by the wider consortium prior to circulation for final approval.

#### Systematic review process

The primary aim of the initial systematic literature review search was to identify which methods and validation protocols are currently used in the scientific literature to validate consumer wearable and smartphone step counting devices. It was not within the scope of this review to examine the validity results of the included studies. The systematic literature review was conducted in line with the Preferred Reporting Items for Systematic Reviews and Meta-Analyses statement and registered with the International Prospective Register of Systematic Reviews (ID: CRD42020177263). Specific search terms were used to identify peer-reviewed journal articles published in three electronic databases: PubMed, Embase and Web of Science. The search terms are presented in online supplemental appendix 1. To be eligible for inclusion, the studies were required to investigate the step count validity of a consumer wearable and/or smartphone, and compare it to a criterion measure such as video recording, visual step counting (ie, laboratory and semifree-living protocols) and/or any device or method including accelerometers, pedometers and wearable garments (ie, free-living protocols). Studies published from inception to 13 March, 2020, across any population, were deemed eligible for inclusion. Studies were excluded if validation of step counting was not an aim, the index device was not a consumer wearable or smartphone, an accelerometer was used as the criterion measure (laboratory studies), a non-research-based accelerometer was used as the criterion measure (semifree-living or free-living studies), the full text was not made available or it was published in the grey literature (eg, congresses, conferences or meeting abstracts). The systematic literature review process was conducted by WJ, PBJ and PMG. Title/abstract screening and full-text screening were completed by two independent reviewers and confirmed by a third independent reviewer using Covidence software (Veritas Health Innovation).^26^ Data were extracted using a standardised template developed by the consortium, allowing for the aggregation of study data under the following headings: sample size included/analysed, sex distribution, type of population, body mass index (BMI), height, weight, age, condition type (laboratory; semifree-living; free-living), criterion measure (description, configuration and placement), index measure (description, configuration and placement), testing protocol, signal processing, data synchronisation, statistical analysis, results and conclusions. The studies were divided into three main condition categories based on the following definitions:

10.1136/bjsports-2020-103147.supp1 Supplementary data

Laboratory: Highly controlled methodologies that involved participants completing gait tasks at controlled or self-selected speeds. This criterion includes studies that require participants to complete gait tasks in indoor and/or outdoor environments, and may require individuals to ambulate on any surface or involve ascent or descent of stairs.

Semifree-living: Semicontrolled methodologies that involve participants completing ‘simulated’ activities of daily living for the purpose of replicating ‘free-living’ conditions (eg, sweeping, cooking, computer use, etc).

Free-living: Uncontrolled methodologies that involve participants wearing the index device during ‘normal’ daily life, outside of a controlled laboratory or simulated environment.

To facilitate evaluation of the current validation protocols, the risk of bias for each article was evaluated under the following headings using the Quality Assessment of Diagnostic Accuracy Studies (QUADAS-2) tool: patient selection, index measure, criterion measure, and study flow and timing.^27^ The methods for the QUADAS-2 evaluation are presented in online supplemental appendix 2.

Additional specific searches of the wider literature were then conducted to help identify and find solutions to address factors that introduce bias during the validation of these devices. For example, identify the optimal walkway length to achieve steady-state walking. The following section presents the current state of knowledge based on the evidence synthesised during literature review process and the INTERLIVE members evidence informed expert opinion.

## Current state of knowledge

**Figure 1:**
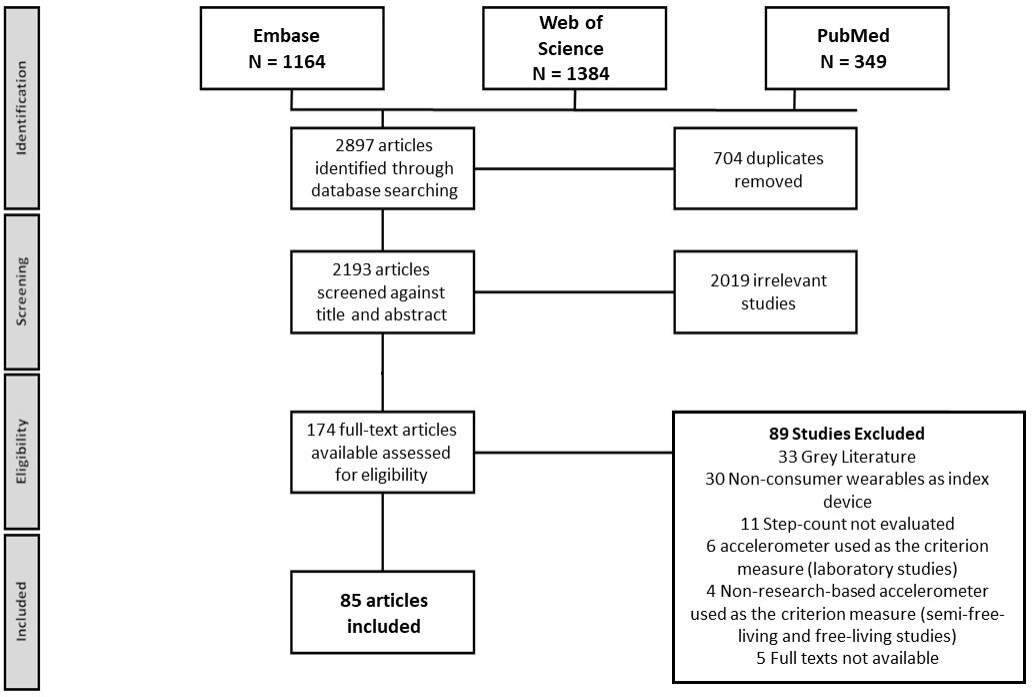
PRISMA flow chart of the systematic review process. PRISMA, Preferred Reporting Items for Systematic Reviews and Meta-Analyses.

The search strategy for this systematic literature review identified 2897 potential articles for inclusion. After the removal of 704 duplicates, 2193 articles were eligible for title and abstract screening. Following this, 174 articles met the criteria for full text screening. Eighty-nine studies were excluded, resulting in a total of 85 articles eligible for the final dataset. Figure 1 illustrates the various stages of the article screening process. Twenty-nine studies investigated the validity of a smartphone-based step counting application, 65 studies investigated a consumer wearable activity tracker, while three studies investigated the validity of an item of smart clothing. Online supplemental appendix 3 (laboratory), online supplemental appendix 4 (semifree-living) and online supplemental appendix 5 (free-living) present the validation methodologies used across the different studies. The risk of bias assessment for each study is presented in online supplementary appendices 6–8. Within our systematic literature review, 98 unique consumer wearables and smartphones were identified, distributed across 50 different brands. From Apple, 11 models of smartphone and 1 smart-watch were included. Nine unique devices were included from Samsung, eight from Fitbit, five from Jawbone, five from Sony, four from Garmin, three from Withings, three from Huawei and two from Omron. An additional 48 unique devices were identified across 45 different brands. The most studied brand was the Fitbit, followed by Apple, Jawbone and Garmin.

**Figure 2:**
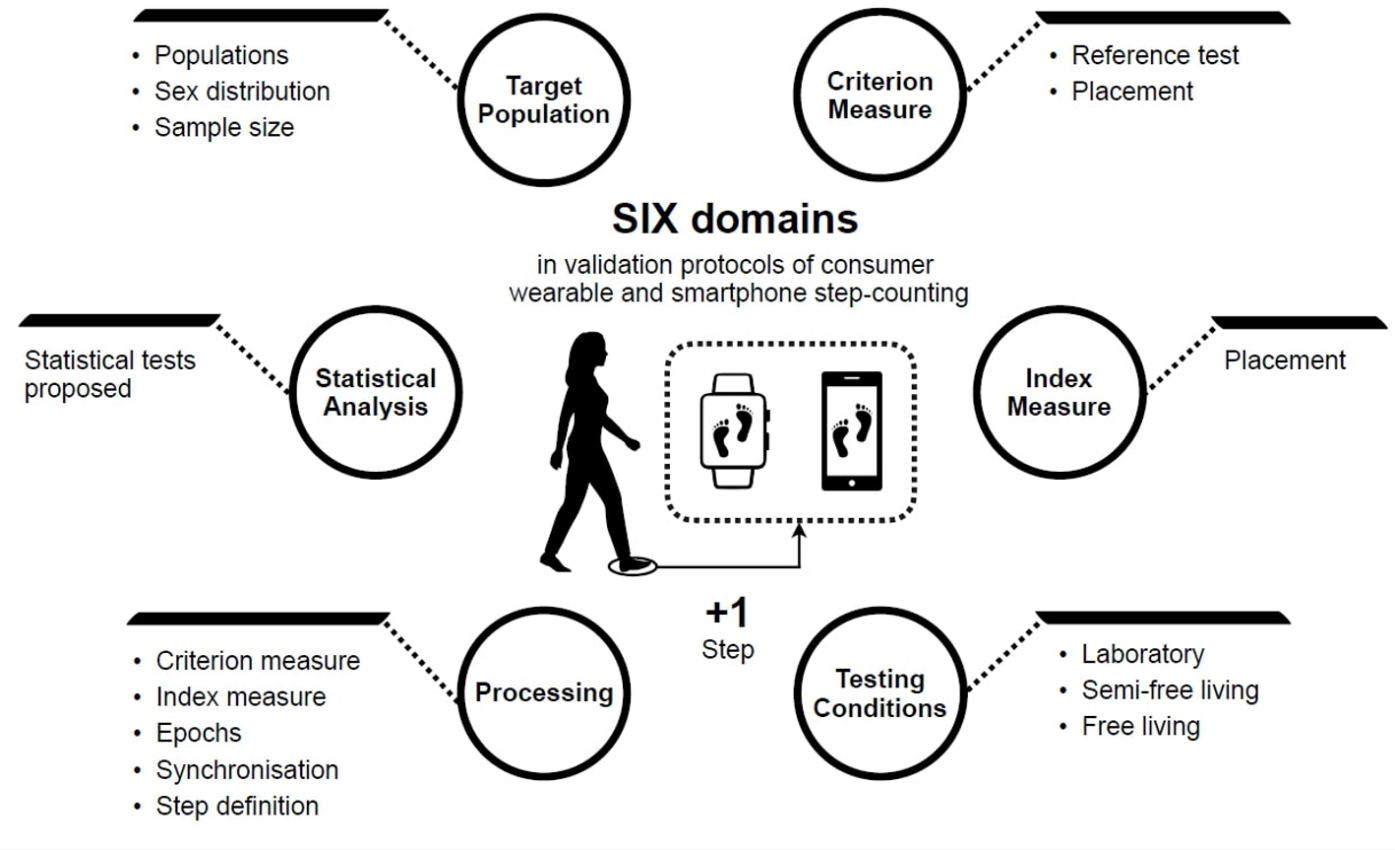
Six domains and corresponding set of key variables of interest which were identified as being of importance when considering validation of consumer wearable and smartphone step counters.

From the synthesised data obtained during the literature review process, the INTERLIVE members identified a set of six key validation protocol and reporting domains: (1) target population, (2) criterion measure, (3) index measure, (4) validation conditions (laboratory, semifree-living and free-living), (5) processing and (6) statistical analysis. A set of key variables of interest were then identified based on an evidence informed expert opinion (figure 2). The following section synthesises the evidence obtained during the statement process and outlines the current state of knowledge regarding validation of consumer wearable and smartphone step counters.

### Target population

**Figure 3:**
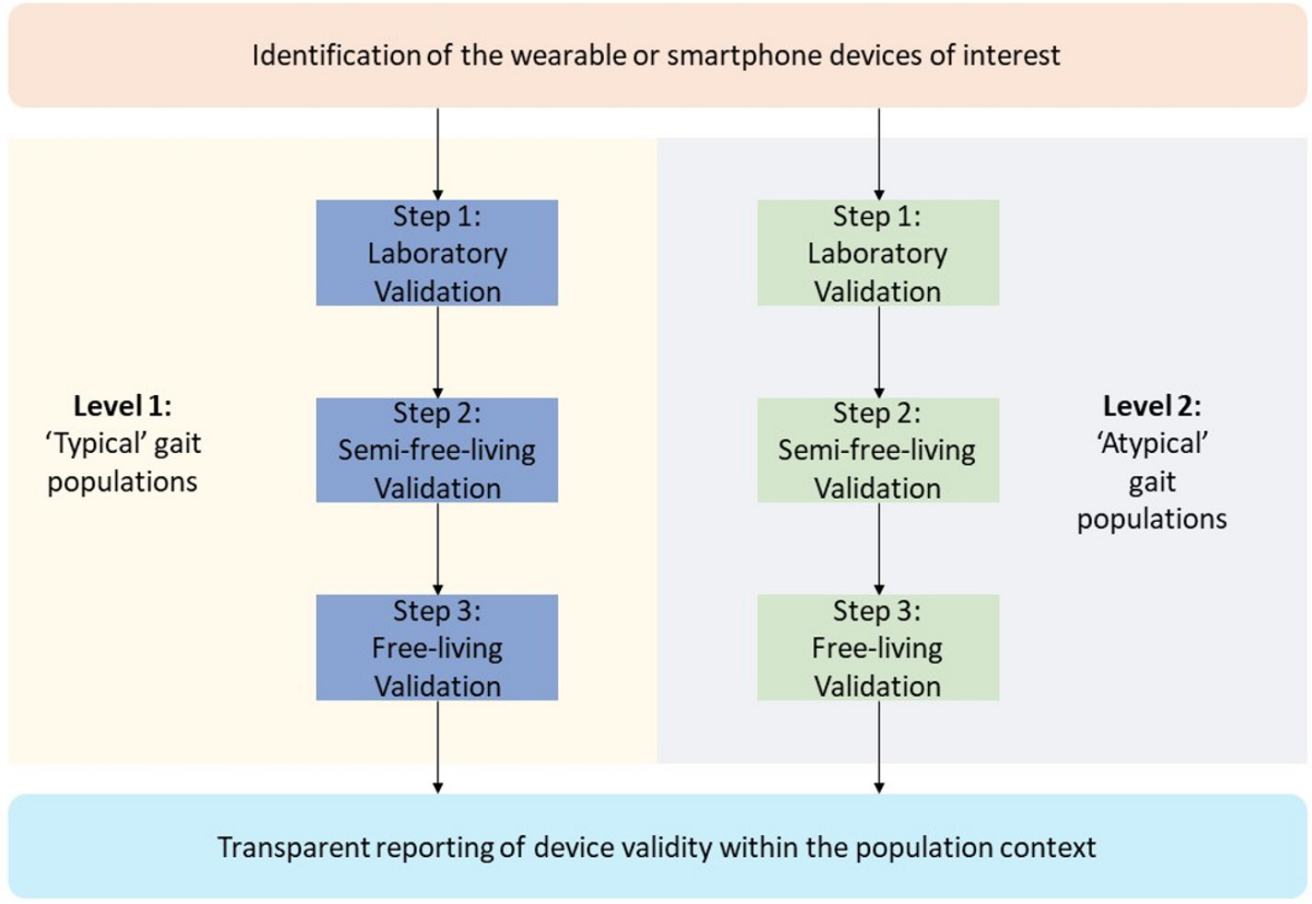
The multistage protocols for the best-practice validation of consumer wearable and smartphone step counters. We recommend a minimum validation in those with typical gait (basic protocol), across laboratory (step 1) and semifree-living (step 2).

It is well understood that gait characteristics are significantly influenced by factors such as physical function,^28^ BMI,^29 30^ age,^31^ and sex.^32 33^ For example, those with Parkinson’s disease walk with a short-stepped, narrow based, shuffling gait, when compared with healthy adults.^34^ Additionally, children and adolescents with obesity have different kinematic and kinetic gait patterns when compared with those with normal weight.^29^ Importantly, if step counting algorithms are developed using solely ‘typical’ gait, the accuracy of wearable and smartphone step counters may be influenced by these different ‘atypical’ gait characteristics. The impact of this has been demonstrated by studies that have highlighted the increased step counting error of these devices at slower walking speeds and in clinical cohorts.^35–38^ To ensure that the validation of the device is representative of the general population, best practice validation should include those with heterogeneous physical function, age and sex. To facilitate this, we propose that those interested in the validation of these step counting devices for use within the wider population should consider separate evaluation of the device within those with ‘typical’ gait characteristics (‘basic’ protocol) and ‘atypical’ gait characteristics (‘advanced’ protocol). Figure 3 provides an overview of the multi-stage nature of the consumer wearable and smartphone step counter validation protocols.

For the basic protocol, recruitment should span three groups: children (<12 years), adolescents/adults (13–64 years) and older adults (≥65 years). The focus of the basic protocol is to validate the step counting device within a ‘typical’ gait population, capturing data from individuals with a range of gait characteristics (eg, speed, cadence, etc). As evidence suggests that gait characteristics stabilise with puberty (12–14 years)^39^ and are altered in older adults,^40^ we recommend to separate recruitment of children, adolescents/adults and older adults. This will help to identify how the device performs across the spectrum of age, among those without ‘atypical’ characteristics that have been shown to affect step counting.

For the advanced protocol, recruitment should focus on validation in populations with ‘atypical’ gait. The focus of this protocol is to introduce factors that are known to negatively affect gait kinematics or wearable/smartphone-based step counting. This will help ensure that the validation process considers the heterogeneous nature of the general population. Examples of the types of populations that should be considered (but not limited to) are those with morbid obesity,^29^ falls-risk,^41^ lower-limb amputations^42^ and neurological conditions.^43^ The validation protocol should be completed within the specific population of interest, and the demographic characteristics of the group should be reported.

Across all studies obtained during the systematic literature review process, the average (range) sample size was 45 (5–592) participants, while authors did not typically complete a formal sample size calculation. Of the 11 studies that conducted formal sample size calculations, the average (range) sample size necessary to reach a power of 0.80 was 42 (16–118) participants. If the focus of the study is to conduct hypothesis testing about a predefined minimal level of accuracy, we advise that a sample size calculation based on previously published or pilot testing mean and SE of the differences between the two devices and predefined clinical maximum allowed difference should be conducted, using the methodologies outline by Lu *et al*.^44^ If sufficient data are not available or this is not the primary focus, based on the average sample size observed in the synthesis of studies which conducted sample size analyses included within the systematic literature review, the observed mean differences and SD of the differences, we advise a minimum of 15 participants for each specific validation group, totalling ≥45 participants, accounting for potential drop-out or data loss.

### Criterion measure

An important consideration during the validation of any device is the validity of the criterion measure to which the index device is being compared. If the criterion measure is not sufficiently valid, then criterion standard bias may be present,^45^ limiting the value that may be gained from such a study. In the context of step counting, across the previously published literature, a total of seven different criterion methods were used. The most common method was a research grade wearable device (n=35 (41%)). This was followed by video recording with one observer (n=23 (27%)), and visual observation with one observer (n=20 (24%)). Only eight (9%) studies used the most rigorous method of video analysis with at least two observers. The most common method under laboratory and semifree-living conditions was video recording, while free-living studies typically used a research grade wearable device (eg, Actigraph, Stepwatch, etc).

Based on the current available evidence, we advise the use of video with two observers for a gold-standard criterion measure of step count. While the use of video recording within a laboratory or semifree-living setting is feasible, the feasibility of using video within a free-living scenario needs to be considered. Previous studies have investigated the feasibility of wearing a body worn camera for 10–14 hours during free-living.^46^ These authors concluded that compliance with the protocol was 87.5%, with participants reporting that they had not changed their behaviour as a result of wearing the device and were not concerned by the amount of wear-time.^46^ From the evaluator’s perspective, feasibility is compromised by the processing time that video recording requires. For instance, a sample size of 45 participants, recorded during 10–14 hours implies 450–630 hours of video to be analysed by two independent observers, which means 4.8–15.8 months of full-time work (8hours/day × 5 days/week × 4 weeks/month) multiplied by two persons, exclusively devoted to counting steps from video recorded. Noteworthy is that these calculations are done for the scenario of a single day (ie, 10–14 waking hours) of video record only, when ideally, free-living validations should be done for a complete week (as usually done in accelerometer-determined PA studies^47^ to cover the interday variability of human activity behaviour. Importantly, the systematic literature review highlighted only one study that has leveraged video recording in free-living conditions.^21^ This investigation^21^ leveraged a waist mounted camera to obtain a gold-standard measurement of step count during free-living activity, over a 24-hour period.

While this approach is undeniably challenging to incorporate into the validation design, it must be acknowledged that currently, no other method has shown sufficient validity to be considered as an alternative gold standard. Despite being commonly used in free-living validation of consumer devices, studies have highlighted that research grade wearable devices are highly sensitive to atypical gait^35–38^ and sensor mounting location.^35^ For example, it has been highlighted that the wrist mounted Actigraph wGT3X+has a high MAPE during slow walking at 1.6 km/hour (wrist 47%) and 3.2 km/hour (wrist 22%). Furthermore, the hip mounting location increased the MAPE to 82% (1.6 km/hour) and 24% (3.2 km/hour), respctivly.^35^ Similarly, the ActivePAL device has been shown to demonstrate increasing step counting error during slower walking speeds.^48 49^ However, this is a rapidly evolving field and despite these current limitations, such approaches are beginning to demonstrate potential. In the aforementioned study,^21^ the StepWatch demonstrated an MAPE of 4% when compared with free-living video in 12 healthy adults with typical gait, highlighting the potential of this approach. However, prior to incorporating such approaches into the validation, we advise that further validation of these potentially ‘equivalent’ criterion methods should be explored, using the validation protocol presented within this statement. The alternative approach should only be considered ‘equivalent’ to the video gold standard if it achieves an MAPE≤5% and there is no evidence of proportional or fixed bias across laboratory, semifree-living, and free-living conditions. In addition, these promising findings should be replicated in future cross-validation studies and if similarly low errors are observed, StepWatch or any other alternative device (accelerometers, insoles, computer vision) could be used as an ‘equivalent’ criterion. Any margin of error should then be reported alongside the study methodology. It is not deemed acceptable to only assess the validity of an alternative criterion device under laboratory or semifree-living, without considering free-living conditions.

### Index measure

An important aspect to consider is the wear location of the wearable or smartphone device during the validation. As the focus of these consumer devices is for unobtrusive use by the general population, it is important for manufacturers and users to ensure that the device is validated in an ecological wear location—the location in which consumers typically use the device. For example, individuals frequently carry smartphones in their pocket,^50–52^ bag^51 53^ or hands,^51 54^ with a cross sectional study highlighting that 60% of females carry their phone in their bag and 60% of men carry their phone in their pocket.^55^ Furthermore, activity trackers are normally worn on the wrist.^56–58^ Conversely, smartphones and wearable activity trackers are not usually attached to the waist,^16 59–61^ hip^62 63^ and chest,^17^ as was observed in several validation studies. Importantly, it has been shown that step counting error is impacted by wear location, with hip-based devices demonstrating a higher accuracy than wrist-worn devices,^64^ while smartphone accuracy changes based on the device, location (waist, arm, hand or bag) and nature of the activity (running or walking).^59 65^ Therefore, it is important to ensure that there is consistency in wear locations within an individual study. We advise that the wear location is specified for each laboratory and semifree-living evaluation. Failure to do so may result in a high degree of inter-subject variability, impacting statistical power and limiting the conclusions that can be drawn from the validation process.

### Testing conditions: laboratory, semifree-living and free-living

The primary function of consumer wearable and smartphone step counters is to provide objective data pertaining to PA, outside of the laboratory environment. However, the systematic literature review highlighted that no single study extensively investigated the validity of the consumer device across controlled laboratory and uncontrolled free-living conditions (online supplemental appendices 3–5). Of the 85 studies included, 57 examined validity using constrained laboratory validation conditions, nine used semifree-living conditions, while 30 used free-living conditions. Two studies used both laboratory and semifree-living conditions, nine used laboratory and free-living conditions, while only one study used semifree-living and free-living conditions. Therefore, the validation protocol proposed within this expert statement has been designed to focus on aspects that have been shown to influence wearable and smartphone step counting algorithms, through a three-stage process.

Laboratory evaluation (stage 1) aims to evaluate the ability of the device to accurately measure steps during self-selected walking and running speeds. This stage focuses on aspects that have previously been shown to negatively affect step counting accuracy, such as short bouts of walking/running, discontinuous walking/running, change of direction, stair climbing and cycling.^66–68^ As previous research has highlighted that 1–7 m is required to enter steady-state-gait,^69^ it is important to ensure that the walkway is sufficiently long. Therefore, as the typical gymnasium is approximately 30 m long (a typical basketball court is 28m × 15m), we advise that the laboratory walking/running trials are conducted using a 30 m long track. Besides validity, an additional evaluation of the within-device precision (reliability) should be investigated during a steady state treadmill walking test. This evaluation should involve two repeated 1 min steady state walking tests, completed at consistent self-selected walking speeds. Previous kinematic research has indicated that footwear^70^ and ground surface type^71 72^ can significantly influence gait kinematics and kinetics. However, a study investigating the impact of these changes on step count accuracy in consumer wearables demonstrated that floor surface (grass, gravel, linoleum, tarmacadam, tile and ramp) did not significantly impact step count accuracy.^73^ Importantly, the authors demonstrated that the step count error may be increased during stair walking (ascending and descending).^73^ Therefore, we advise that the laboratory validation is conducted on both flat surfaces and during stair-walking. If the investigators are particularly interested in the validation of the device on alternative surfaces we advise that the laboratory protocol is conducted on relevant alternative surfaces (grass, gravel, linoleum, tarmacadam, tile, incline/decline, etc) to ensure ecological validity.

The semifree-living evaluation (stage 2) aims to examine the performance of the step counters during simulated sedentary, household and exercise-based activities of daily living. It is well established that activities of daily living frequently result in the false detection of steps by wearable and smartphone devices. A previous study^66^ demonstrated that non-stepping activities such as deskwork, taking an elevator, taking a bus journey, driving an automobile, washing and drying dishes, functional reaching tasks, indoor cycling, outdoor cycling and indoor rowing often result in the detection of false steps. While there is evidence^74^ suggesting that monitors may underestimate steps by 35%–64% during household tasks. The proposed sedentary, household and exercise-based tasks are among the most common PAs in both youth and adulthood, covering a wide spectrum of the human movement pattern.^75 76^ For instance, we include different positions (lying, sitting and standing), fine and gross motor control tasks such as writing or rowing exercises respectively and the combination of steady state situations (eg, standing and talking) with locomotion activities (eg, simulated shopping). We also include a considerable range of intensities according to the compendium of PAs, which is relevant in terms of step detection since some wearables have demonstrated a weak performance in low intensity activities.^35^ Activities that involve arms and hands movements have registered a great number of false steps, and therefore, we propose the inclusion of arm-movement activities not including steps (eg, washing dishes) and including steps (eg, vacuuming).^57 66 77^ Ultimately, the number of potentially valuable simulated activities to include is endless; however, from a pragmatical point of view, the protocol we propose includes a selection of relevant activities that can be performed in less than 45 min. The incorporation of a free-living validation component will serve to ensure that a wide range of activities that have not been considered in laboratory conditions are included.

The free-living evaluation (stage 3) aims to capture a wide array of activities of daily living ‘in the wild’, accounting for the differences observed between controlled and uncontrolled activities. Evidence suggests that individuals typically have different gait characteristics during laboratory and free-living assessments.^28^ The impact of this is highlighted by the increased wearable and smartphone step counting error observed during free-living conditions,^21^ when compared with laboratory conditions.^20^ We advise that during this stage, participants are encouraged to engage in their usual activities of daily living, to quantify measurement error in an unconstrained environment, over a period of at least 24 hours, but ideally 1 week. A week long recording period, resulting in a minimum of 3–4 days of recorded time is optimal to capture interday and weekday-weekend day variation.

### Processing of index and criterion data

#### Synchronisation between criterion and index measures

When comparing a method of measurement to a gold-standard criterion, an important aspect that should not be overlooked is the development of a precise synchronisation methodology to align the data captured from the index and criterion. While the methods used to synchronise the measures may seem trivial to some, if not done correctly, any discrepancy may introduce error, biasing the results. For example, the step count on a device may be ‘contaminated’ by activity that took place before or after the activity recorded by the criterion measure.

We advise that adequate synchronisation is achieved by requiring participants to stand still at the beginning and the end of all trials while the step count and video time are noted. This pragmatic approach should be used across laboratory, semifree-living, and free-living conditions, and may be specific to the index device of interest. For example, some devices may allow step count to be recorded from the device interface, while others may require synchronisation with a smartphone before it can be viewed.^66^

If an alternative ‘equivalent’ criterion measure to video is being used, then the time-stamp and/or step count should be noted at the beginning and the end of each trial for both measures. In addition, the synchronisation method should be reported in detail within the validation report to ensure transparency and replicability.

#### Criterion device step counting

If video is used as the criterion measure, to ensure gold-standard validity, ≥2 observers should view each video in real-time and count the number of steps using a manual counting device. During periods of uncertainty, where it is not clear if a step took place, the video should be reviewed in slow motion. The systematic literature review highlighted eight studies that used ≥2 observers counting the number of steps.^37 78–84^ The observers should be sufficiently trained to ensure excellent agreement between reviewers and the interobserver reliability should be reported.^37 80 82^ If an equivalent criterion method is being used, the data processing methods that have been described within the previously published validation study should be followed.

An important aspect to consider in the validation of these step counting devices is what constitutes a ‘step’. Typically, a step is defined in biomechanical terms as the period during the gait cycle between two consecutive heel strikes^85^ or in more general terms as ‘the act of putting one leg in front of the other in walking or running’. 86 However, these definitions are not suitable for this context as they are either too constrained (biomechanical definition) or do not provide sufficient guidance for deciding what constitutes a step within this context (locomotion definition). Furthermore, these definitions disregards activities of daily living that involve steps but without a locomotive purpose, such as vacuuming or hanging out clothes. Therefore, for the purpose of validating wearable and smartphone step counters that are designed for the purpose of quantifying PA and energy expenditure, the INTERLIVE network has defined a step for the as follows:

‘The act of raising one foot and putting it down in another spot, resulting in the displacement of the centre of mass’.

This definition will allow for step counting during continuous locomotion (walking/running), as well as during intermittent gait, common during activities of daily living.

### Statistical analysis

In order to thoroughly consider the validity of an index device when compared with a criterion measure, agreement between the two devices should be evaluated.^87^ Agreement provides details about how accurate a measurement tool is when compared with a reference standard.^88^ A systematic literature review of statistical methods employed in medical instrument validity studies highlighted that a large proportion of studies have used inappropriate statistical methods.^89^ In line with these conclusions, many of the studies included within our systematic literature review inappropriately used measures of relative measurement reliability (ie, intraclass correlation coefficient, kappa, etc) (n=37 (44%)), correlation (ie, Pearson, Spearman, etc) (n=34 (40%)), and comparison of means (n=33 (39%)).

To thoroughly investigate validity, we advise the use of Bland-Altman limits of agreement analysis and MAPE. Bland-Altman analysis is widely accepted as the most appropriate tool in assessing agreement within medical validation studies, providing a measure of the agreement between the two measurements.^87 89^ This method was the most common approach seen across the previously published studies (n=43 (51%)). To investigate the presence of any proportional or fixed bias, least-products regression should be used as part of the Bland-Altman analysis.^90^ To facilitate comparison between devices and testing conditions, an absolute measure of error, such as MAPE should be used. MAPE is the average of the absolute percentage of error of a tool and is commonly used to describe the error of a prediction model,^91^ and was used by 28 studies within our review (33%). Together, these statistical approaches provide a means to thoroughly evaluate the validity of the wearable and smartphone step counters and help identify the presence of any measurement bias.

The within-device precision (reliability) of the index device should be investigated by comparing the total number of steps obtained during the repeated 1 min steady state treadmill walking tests, using intra-class correlation coefficient with 95% CIs.

When considering what constitutes a valid device, readers should consider the results within the context of the potential use case. Specifically, if an activity tracker is to be used as an outcome measure within a clinical trial or as an alternative gold-standard measurement tool for step counting, we recommend that the device should demonstrate an extremely low level of measurement error (MAPE ≤5%) and bias, within the clinical population of interest. Conversely, if the device is being validated for use by the general population, then we recommend that a marginally higher error (MAPE ≤10%–15%) may be acceptable. Therefore, if the goal of the evaluation is not to conduct hypotheses testing and a formal sample size calculation has not been performed, binary conclusions about the validity should not be made. Rather, the level of measurement error and bias should be openly reported, taking into consideration all the proposed validation conditions (laboratory; semifree-living; free-living), to facilitate contextual interpretation.

## Recommended validation protocol

Based on the available evidence outlined within this statement, INTERLIVE recommends that wearable and smartphone step counter manufacturers seek to validate their devices using standardised and transparent methodologies. We advise that manufacturers engage in a multistage validation protocol. Broadly, this protocol examines the validity of these step counter devices under laboratory (stage 1), semifree-living (stage 2) and free-living conditions (stage 3), across populations with ‘typical’ (basic protocol) and ‘atypical’ (advanced protocol) gait characteristics. The basic protocol should be used by those interested in demonstrating the validity of step counting devices within populations that do not demonstrate ‘atypical’ gait characteristics known to influence wearable and smartphone based step counting. If the goal of the evaluator is to demonstrate the validity of the device across the wider population in those with known ‘atypical’ gait characteristics, the advanced protocol should also be used. Figure 3 provides an overview of the multistage nature of the consumer wearable and smartphone step counter validation protocols. The proposed framework sets out to ensure that the recruited target population is fit-for-purpose, addressing many of the sources of bias identified during the review of the literature. Table 1 presents a detailed best-practice validation protocol, while table 2 presents a check-list designed to facilitate validation protocol planning. In addition, a comprehensive protocol reporting sheet for use by both research institutions and industry is presented within table 3 to facilitate standardised and transparent data sharing. INTERLIVE advises that wearable and smartphone step counter manufacturers seek to achieve the basic and advanced multistage validation protocols, completing the laboratory, semifree-living and free-living validation (stages) across populations with ‘typical’ (basic protocol) and ‘atypical’ (advanced protocol) gait characteristics. However, in the scenario where it is not possible to perform the whole validation protocol, at a minimum, the basic laboratory (stage 1) and semifree-living (stage 2) validation, within a ‘typical’ gait population should be achieved.

**Table 1:** The proposed best-practice protocols for the validation of wearable and smartphone step counting devices

Methodological domains	Methodological variables	Protocol considerations	Reporting considerations
1. Target population	**1.1. Population**	Cross section of participants across the spectrum of ages*, **Three groups:** *1*. Children (<12 years) *2*. Adolescents and healthy adults (13–64 years) 3. Older adults (>65 years) * The basic validation protocol can be performed in healthy participants without any specific disease or condition that can impact a typical gait. The advanced protocol should be completed in special populations with atypical gait characteristics, such as morbid obesity, falls-risk, lower-limb amputees and neurological conditions. In the advanced protocol, participants may use any walking aid if required.	Provide detailed demographics for each group (ie, age, height, weight, BMI, health condition). Report means and ranges.
	**1.2. Sex**	Equal Sample of males and females in each group.	Report the sex distribution in each group.
	**1.3. Sample size**	If the focus of the study is to conduct hypothesis testing about a predefined minimal level of accuracy, a sample size calculation should be completed based on the previously published or pilot study mean and SE of the differences between the devices, using the methodologies outline by Lu *et al*.^44^ If sufficient data are not available or this is not the focus of the evaluation, we advise a minimum of 15 participants for each specific validation group (i.e. ≥45 participants).	Detail sample recruited and sample analysed for all groups and in all levels of the study. If a sample size calculation is used, provide details of the assumptions.
2. Criterion measure	**2.1. Reference test**	Video camera with multiple observer (≥2) or equivalent*. Test the agreement between observers. * Any device or method which has been demonstrated to possess less than 5% measurement error using the laboratory, semifree-living, and free-living validation protocols detailed below. Additionally, this should be specific to the population of interest.	Report camera setup and the level of agreement between observers. If an equivalent method is used, its validity must be reported.
	**2.2. Placement**	**Laboratory and Semifree-living:** Ensure the whole scene is in the field of view. **Free-living:** Camera should be affixed to the body in a manner that does not affect gait pattern, activities of daily living, and allows the evaluator to see the step field of view. For example, attached to a belt around the waist. If an ‘equivalent’ criterion is used, the device should be used as per the methods described within the validation study.	Describe in detail the setup including special situations (eg, going to the toilet).
3. Index measure	**3.1 Placement**	Should be placed in an ecological body location which the consumer device was designed for. **Smartphones:** clothing pockets, hand-held, handbags or purses, belt phone holder. Phone should not be mounted to the body in an unnatural way (eg, strapped to chest etc). **Wearable Activity Monitor:** should be worn as per the manufacturer’s instructions.	Report the exact model and version for the index measure including hardware and software and report in detail the placement protocol.
4. Testing conditions	4.1. Laboratory assessment protocol	**Walking:** Self-selected walking speed. 3 min walk test (30 m walkway) 3 min zig-zag walk test (30 m walkway - three cones) 3 x stair test ascent/descent (minimum 12 step staircase) 3 min stationary cycling 2×1 min steady state treadmill test (consistent, self-selected walking speed). **Running/fast-walking:** Self-selected running speed. 3 min run test (30 m walkway) 3 min zigzag run test (30 m walkway - three cones) **Optional:** Evaluation on alternate floor surfaces (grass, gravel, linoleum, tarmacadam, tile, incline/decline etc). **Estimated time:**~25 min	Report cadence/ gait velocity for each trial (eg, 3 min walk test) and for each group (eg, children) Report the overground surface and footwear condition being used. If possible, report the air temperature
	**4.2 Semifree-living assessment protocol**	Participants are encouraged to complete the tasks as they would do during activities of daily living, at a self-selected pace. Each activity should be completed for ≥3 min unless otherwise stated. **Sedentary activities:** simulated sleeping (Get into bed, after 1 min roll over, after 2 min check time on smartphone/wearable, after 3 min get up) writing by hand (sitting) eating/ drinking (sitting) computer use (both typing and mouse use) smartphone use (sitting) simulated video-game (game console controller) **Household activities:** standing and talking sweeping (5 m squared) vacuuming (5 m squared) folding laundry (no steps while standing) simulated washing/drying dishes (washing for 1.5 min and drying for 1.5 min) simulated shopping (pick up two shopping bags, walk 10 m, empty bag and put items in cupboard—perform task once) **Exercise related activity:** Exercise difficulty should be selected by the evaluator based on the participants ability. Squat/sit-to-stand (≥20 s) Lunge/split-squat (≥20 s) Low rowing exercise (≥20 s) **Estimated time:**~45 min	Describe in detail the setup for each trial (eg, computer use) to ensure transparency. Report the overground surface being used. If possible, report the air temperature
	**4.3 Free-living assessment protocol**	≥24 hours free-living period whereby the participant completes activities of daily living in an unconstrained environment (home, work, travelling, etc). A weeklong recording period is optimal to capture interday and weekday-weekend day variation.	Describe in detail the setup to ensure transparency.
5. Processing	5.1 Criterion measure processing *	The recorded video should be reviewed by >2 independent reviewers in real time and a counting device should be used to record steps. * Periods of uncertainty, where it is not clear if a step took place, should be reviewed in slow-motion.	**Camera:** Report camera frame rate and agreement between reviewers. Equivalent: report all processing methods in detail.
	**5.2 Index measure processing**	Ensure alignment of epochs from criterion measure and index device.	Detail the index device processing as much as possible.
	**5.3 Epochs for analysis**	**Laboratory and Semifree-living:** Step counts, and video times should be noted after each trial and analysed for each component of the protocol. Steps which occur between trials should be discarded. Cadence (steps/min) and gait velocity (m/s) should be calculated from the video data. **Free-living:** Step counts and video times should be noted by the evaluator at the beginning and end of the assessment period (≥24 hours). Step counts should be analysed for the full period. Where available, step counts should be divided into shorter hour by hour epochs to facilitate a more granular analysis (eg, hour-by-hour accuracy or activity-by-activity accuracy). This is dependent on the capabilities of the index device.	**Laboratory and semifree-living:** Report error for the overall protocol and individually for each trial. **Free-living:** Report the valid wear time for comparison and the error for the entire period or a more granular analysis if available.
	**5.4 Index and criterion synchronisation**	**Laboratory and semifree-living:** Participants should stand still at the beginning and end of each activity trial while the step count and video time are noted. **Free-living:** Stand still at the start of the period and film the number of steps recorded on wearable/smartphone. Complete the wear period. Stand still at the start of the period and film number of steps recorded on wearable/smartphone.	Describe in detail the synchronisation process allow replication.
	**5.5 Step definition**	The act of raising one foot and putting it down in another spot, resulting in the displacement of the centre of mass.	Clearly report the adopted definition.
6. Statistical analysis	**6.1 Statistical tests**	**Device Accuracy** Bland-Altman with LoA Least products regression of the differences against the means MAPE **Device precision** Intraclass correlation with 95% CIs (calculated for the 2×1 min treadmill test only)	Unless a formal sample size analysis has been conducted, binary conclusions about the validity should not be made. Rather, the level of measurement error and bias should be openly reported, taking into consideration all the proposed validation conditions (lab; semi; free-living), to facilitate contextual interpretation.

If the evaluator decides not to perform the whole validation protocol, there is a minimal protocol to be followed. At a minimum, the validity should be investigated in those with typical gait (basic protocol), across laboratory (step 1) and semifree-living (step 2). Observers should determine which participants are deemed suitable for the different components of the assessment protocols.

*Any device or method which has been demonstrated to possess less than 5% measurement error using the laboratory, semifree-living, and free-living validation protocols detailed below. Additionally, this should be specific to the population of interest. For the case of free-living validation, the INTERLIVE network is aware that video recording and ‘manual’ step counting by two independent evaluators over ≥24 hours recording period (a 1-week recording period (minimum 3–4 complete days) is optimal to capture interday and weekday-weekend variation) is extremely costly in time and resources and therefore likely not feasible for many. Therefore, we feel that this field needs to move forward, developing and validating alternative methods such as insole sensors that after validation and cross-validation could be used as a new and more feasible gold-standard method for free-living validation protocols.

BMI, body mass index; INTERLIVE^®^, Intelligent Health and Well-Being Network of Physical Activity Assessment; LoA, limits of agreement; MAPE, mean absolute percentage error.

**Table 2:** Check-list of items to be considered during the validation protocol development for consumer wearable and smartphone step counters

Target population assessment	
Age	
(Children (<12 years)	◯
Adolescents and healthy adults (13–64 years)	◯
Older adults (>65 years)	◯
Sex (equal sample of males and females)	◯
Sample size calculation via pilot study OR Sample of convenience (n≥45)	◯
**Criterion measure assessment**	
Video camera with multiple observers (≥2) or equivalent*	◯
Placement to ensure steps are within the field of view†	◯
**Index device assessment**	
Placement according to manufacturer’s instructions	◯
**Laboratory testing condition assessment**	
Walking:	
3 min walk test	◯
3 min zigzag walk test	◯
3 x stair test ascent/descent	◯
3 min stationary cycling	◯
2×1 min steady state treadmill test (reliability)	◯
Running/fast-walking:	
3 min run test	◯
3 min zig-zag run test	◯
Optional	
1 x incline/ decline walking test	◯
**Semifree-living testing condition assessment**	
Sedentary activities:	
Simulated sleeping	◯
Writing by hand	◯
Eating/ drinking	◯
Household activities:	
Standing and talking	◯
Sweeping	◯
Vacuuming	◯
Folding laundry	◯
Simulated washing/drying dishes	◯
Simulated shopping	◯
Exercise-related activities:	
Squat/sit-to-stand	◯
Lunge/split-squat	◯
Low rowing exercise	◯
**Free-living testing condition assessment**	
Subject’s wear index and criterion device for a minimum of 24 hours, and if a more feasible gold-standard or pseudo-gold-standard method is developed/validated a week assessment would be ideal.	◯
**Processing**	
Criterion measure processing	
3. **Video**: Recorded video should be reviewed by >2 independent observers in real time and a counting device should be used to record steps OR **Equivalent:** automated method which has previously demonstrated sufficient validity (* see footnote)	◯
Index measure processing	
No post processing of the end-user data is allowed	◯
Epochs for analysis	
Note step count at start and end of each trial	◯
Discard steps which occur between trials	◯
Record cadence and gait velocity for laboratory and semifree-living	◯
Index and criterion synchronisation	
Participants stand still at start and end of each activity trial while step count and video time (or equivalent criterion count) are noted Step definition	◯
The act of raising one foot and putting it down in another spot, resulting in the displacement of the centre of mass	◯
**Statistical analysis**	
Mean difference or mean relative difference Bland-Altman LoA	◯
Least products regression of the differences against the means	◯
Mean absolute percentage error	◯
Intraclass correlation coefficient (calculated for the 2×1 min treadmill test only)	◯

*Any device or method which has been demonstrated to possess less than 5% measurement error using the laboratory, semifree-living, and free-living validation protocols detailed below. Additionally, this should be specific to the population of interest. For the case of free-living validation, the INTERLIVE network is aware that video recording and ‘manual’ step counting by two independent evaluators over ≥24 hours recording period (a 1-week recording period (minimum 3–4 complete days) is optimal to capture interday and weekday-weekend variation) is extremely costly in time and resources and therefore likely not feasible for many. Therefore, we feel that this field needs to move forward, developing and validating alternative methods such as insole sensors that after validation and cross-validation could be used as a new and more feasible gold-standard method for free-living validation protocols.

†If an ‘equivalent’ criterion is used, the device should be used as per the methods described within the validation study.

INTERLIVE^®^, Intelligent Health and Well-Being Network of Physical Activity Assessment; LoA, limits of agreement.

**Table 3:** Minimum required reporting sheet for standardised and transparent data sharing

Target population	Description	Reporting
Sampling method	Random, convenient, etc.	
Distribution of sex	♂=n/♀=n	
Age	Mean±SD and range (years)	
BMI	Mean±SD and range (kg/m²)	
Sample size	Provide the no and explain how the sample size was chosen	
Health condition (where relevant)	Provide detailed description of cohort with atypical gait characteristics	
**Criterion measure**		
Video camera with multiple observer (≥2) or equivalent*	**Video:** Model and brand OR **Equivalent:** Model, brand and published measurement error	
Placement	Actual placement of camera or equivalent	
**Index device**		
Placement	Manufacturer’s instructions and actual placement	
**Testing protocol**		
Type of protocol	Laboratory, semifree-living and/or free-living	
Type of trial	List the components of the recommended protocol evaluated (eg, laboratory 3 min walk test, 3 min zig-zag walk test etc)	
Duration	Duration of each trial	
Contextual factors	Where the testing took place, weather conditions, time of year	
**Processing**		
Criterion measure processing	**Video**: step definition; methods for reviewing video; number of raters OR **Equivalent:** processing method for equivalent criterion	
Index measure processing	Detail methods if end-user data is processed (eg, excluded data etc.)	
Epochs for analysis	In seconds (where relevant)	
Index and criterion synchronisation	Method used for synchronising step data from index and criterion measures	
**Statistical Analysis assessment**		
Mean difference or mean relative difference Bland-Altman LoA	Detail the LoA for each trial	
Least products regression of the differences against the means	Detail the presence of any systematic or proportional bias for each trial	
MAPE	Detail the MAPE for each trial	
Intraclass correlation coefficient	Detail the intra-class correlation coefficient and 95% CI for the 2×1 min treadmill test	

*Any device or method which has been demonstrated to possess less than 5% measurement error using the laboratory, semi free-living, and free-living validation protocols detailed below. Additionally, this should be specific to the population of interest. For the case of free-living validation, the INTERLIVE network is aware that video recording and ‘manual’ step counting by two independent evaluators over ≥24 hours recording period (a 1-week recording period (minimum 3–4 complete days) is optimal to capture interday and weekday-weekend variation) is extremely costly in time and resources and therefore likely not feasible for many. Therefore, we feel that this field needs to move forward, developing and validating alternative methods such as insole sensors that after validation and cross-validation could be used as a new and more feasible gold-standard method for free-living validation protocols.

BMI, body mass index; INTERLIVE^®^, Intelligent Health and Well-Being Network of Physical Activity Assessment; LoA, limits of agreement; MAPE, mean absolute percentage error.;

## Discussion and future directions

This INTERLIVE expert statement strives to provide actionable and unambiguous recommendations for those interested in the thorough and transparent validation for consumer wearable and smartphone step counters. Based on the best available evidence, a detailed validation protocol has been presented under the domains of target population, criterion measure, index measure, validation conditions (laboratory, semifree-living and free-living), processing and statistical analysis.

It is clear that past approaches used by industry to validate wearable and smartphone step counters are not fit-for-purpose. While early attempts by the CTA to develop validation standards were a step in the right direction, the CTA validation approach did not address many of the potential sources of bias that have been highlighted above and has not been widely adopted.^19^ As such, the decision on device validation lies with the manufacturer, with no regulatory or certification standards currently in place to ensure transparency and scientific rigour. This means that there is a high degree of heterogeneity across the methodologies used to validate these devices, with no means for interested stakeholders to objectively compare devices. A rigorous, standardised and transparent validation process should be the mutual interest of manufactures, scientific institutions and consumers in order to judge whether these devices are useful and perform with satisfactorily low measurement error. The approach presented within this statement will help progress towards the goal of ‘evidence-based marketing claims’, ensuring that wearable technology can be used safely and to its full potential.^18^ It is intended that the recommendations outlined within this statement provide an evidence-based means for manufacturers to ensure that their devices are validated to an acceptable standard, using rigorous and transparent scientific methods. As a next step, INTERLIVE plans to provide best-practice recommendations for validation of wearable and smartphone based energy expenditure and maximal oxygen consumption (VO_2_ max) estimations, contributing to the development of accepted standards for the validation of wearable and smartphone PA tracking devices.

If the standardised validation of these devices can be realised, there are many potential positive implications for all stakeholders. Manufacturers will be able to transparently communicate the value of their devices to consumers. Furthermore, transparency will drive innovation, as the industry strives to achieve improved validity. General consumers will be able to compare the validity of potential devices, ensuring that they can make an informed decision about the most appropriate device for them. Similarly, this will improve transparency for healthcare providers who are interested in incorporating these devices into every day clinical practice, facilitating evidence based digital healthcare. Finally, scientific researchers will be able to understand the validity of these devices, facilitating progress in the realms of ‘passive’ digital phenotyping and ‘active’ PA behavioural change research. As such, we urge all stakeholders to engage in the development and validation of these devices to adhere to these best-practice recommendations.

The thorough validation of these devices does not come without challenges. The systematic literature review process highlighted a wide range of criterion methods used to count steps. Despite this, we advise that there is currently not sufficient evidence to support the use of alternatives to video recording as the gold-standard measurement of step count. However, as mentioned above, there are significant challenges associated with the use of video analysis within the free-living environment. Therefore, we recommend that researchers and manufacturers should focus on the development and thorough validation of alternatives that may improve the practicality of free-living validation. Potential approaches that are beginning to demonstrate promise are the use of research grade accelerometers placed at the ankle or foot (for closer relation to the foot stepping vs the usual placements of hip or wrist),^21 92^ insoles^93^ and machine learning-based video analysis.^60^ If proven to be sufficiently accurate when compared with video-based step count, these approaches may serve to improve the feasibility of free-living validation studies.

## Conclusions

This INTERLIVE expert statement provides an evidence-informed best-practice consumer wearable and smartphone step counter validation protocol. The systematic literature review included within, highlighted a high degree of heterogeneity between previously published methods, with many studies failing to address key sources of validation bias. The INTERLIVE consortium recommend that the proposed validation protocol should be used when considering the validation of any consumer wearable or smartphone step counter to overcome the main identified issues. The consortium also provide guidance for future research activities, highlighting the imminent need for the development of feasible alternative ‘gold-standard’ criterion measures for free-living validation. Adherence to this validation standard will help ensure methodological and reporting consistency, facilitating comparisons between consumer devices and the amalgamation of standardised open datasets. This will ensure that manufacturers, consumers, healthcare providers and researchers can use this technology safely and to its full potential.

## References

[1] Liu S . Fitness & activity tracker - Statistics & Facts Statista2019, 2020. Available: https://www.statista.com/topics/4393/fitness-and-activity-tracker/ [Accessed 12 May 2020].

[2] O'Dea S . Global smartphone sales to end users 2007-2021, 2020. Available: https://www.statista.com/statistics/263437/global-smartphone-sales-to-end-users-since-2007/ [Accessed 30 Oct 2020].

[3] Evenson KR , Goto MM , Furberg RD . Systematic review of the validity and reliability of consumer-wearable activity trackers. Int J Behav Nutr Phys Act 2015;12:159. 10.1186/s12966-015-0314-1 26684758 PMC4683756

[4] Brickwood K-J , Watson G , O'Brien J , et al . Consumer-Based wearable activity Trackers increase physical activity participation: systematic review and meta-analysis. JMIR Mhealth Uhealth 2019;7:e11819. 10.2196/11819 30977740 PMC6484266

[5] Kraus WE , Janz KF , Powell KE , et al . Daily step counts for measuring physical activity exposure and its relation to health. Med Sci Sports Exerc 2019;51:1206–12. 10.1249/MSS.0000000000001932 31095077 PMC6527133

[6] Saint-Maurice PF , Troiano RP , Bassett DR , et al . Association of daily step count and step intensity with mortality among US adults. JAMA 2020;323:1151–60. 10.1001/jama.2020.1382 32207799 PMC7093766

[7] Lee I-M , Shiroma EJ , Kamada M , et al . Association of step volume and intensity with all-cause mortality in older women. JAMA Intern Med 2019;179:1105–12. 10.1001/jamainternmed.2019.0899 31141585 PMC6547157

[8] Hansen BH , Dalene KE , Ekelund U , et al . Step by step: association of device-measured daily steps with all-cause mortality-A prospective cohort study. Scand J Med Sci Sports 2020;30:1705–11. 10.1111/sms.13726 32427398 PMC7496562

[9] Hall KS , Hyde ET , Bassett DR , et al . Systematic review of the prospective association of daily step counts with risk of mortality, cardiovascular disease, and dysglycemia. Int J Behav Nutr Phys Act 2020;17:78. 10.1186/s12966-020-00978-9 32563261 PMC7305604

[10] Okwose NC , Avery L , O’Brien N , et al . Acceptability, feasibility and preliminary evaluation of a novel, personalised, home-based physical activity intervention for chronic heart failure (Active-at-Home-HF): a pilot study. Sports Med Open 2019;5:45 10.1186/s40798-019-0216-x 31776701 PMC6881484

[11] Althoff T , Sosič R , Hicks JL , et al . Large-Scale physical activity data reveal worldwide activity inequality. Nature 2017;547:336–9. 10.1038/nature23018 28693034 PMC5774986

[12] Evidation . COVID-19 pulse: delivering regular insights on the pandemic from a 150,000+ person connected cohort. Evidation, 2020.

[13] Van Remoortel H , Giavedoni S , Raste Y , et al . Validity of activity monitors in health and chronic disease: a systematic review. Int J Behav Nutr Phys Act 2012;9:84. 10.1186/1479-5868-9-84 22776399 PMC3464146

[14] Straiton N , Alharbi M , Bauman A , et al . The validity and reliability of consumer-grade activity trackers in older, community-dwelling adults: a systematic review. Maturitas 2018;112:85–93. 10.1016/j.maturitas.2018.03.016 29704922

[15] Arch ES , Sions JM , Horne J , et al . Step count accuracy of StepWatch and FitBit One™ among individuals with a unilateral transtibial amputation. Prosthet Orthot Int 2018;42:518–26. 10.1177/0309364618767138 29623810 PMC12166528

[16] Brodie MA , Pliner EM , Ho A , et al . Big data vs accurate data in health research: large-scale physical activity monitoring, smartphones, wearable devices and risk of unconscious bias. Med Hypotheses 2018;119:32–6. 10.1016/j.mehy.2018.07.015 30122488

[17] Ebara T , Azuma R , Shoji N , et al . Reliability of smartphone-based gait measurements for quantification of physical activity/inactivity levels. J Occup Health 2017;59:506–12. 10.1539/joh.17-0101-OA 28835575 PMC5721272

[18] Sperlich B , Holmberg H-C . Wearable, Yes, but able…?: it is time for evidence-based marketing claims! Br J Sports Med 2017;51:1240. 10.1136/bjsports-2016-097295 27986762 PMC5537552

[19] CTA . Physical activity monitoring for step counting, 2016. Available: www.cta.tech

[20] Fokkema T , Kooiman TJM , Krijnen WP , et al . Reliability and validity of ten consumer activity Trackers depend on walking speed. Med Sci Sports Exerc 2017;49:793–800. 10.1249/MSS.0000000000001146 28319983

[21] Toth LP , Park S , Springer CM , et al . Video-Recorded validation of wearable step counters under free-living conditions. Med Sci Sports Exerc 2018;50:1315–22. 10.1249/MSS.0000000000001569 29381649

[22] Bassett DR , Rowlands A , Trost SG . Calibration and validation of wearable monitors. Med Sci Sports Exerc 2012;44:S32–8. 10.1249/MSS.0b013e3182399cf7 22157772 PMC3273335

[23] Keadle SK , Lyden KA , Strath SJ , et al . A framework to evaluate devices that assess physical behavior. Exerc Sport Sci Rev 2019;47:206–14. 10.1249/JES.0000000000000206 31524786

[24] Freedson P , Bowles HR , Troiano R , et al . Assessment of physical activity using wearable monitors: recommendations for monitor calibration and use in the field. Med Sci Sports Exerc 2012;44:S1–4. 10.1249/MSS.0b013e3182399b7e 22157769 PMC3245520

[25] Welk GJ , McClain J , Ainsworth BE . Protocols for evaluating equivalency of accelerometry-based activity monitors. Med Sci Sports Exerc 2012;44:S39–49. 10.1249/MSS.0b013e3182399d8f 22157773

[26] Veritas . Covidence systematic review software veritas health innovation, 2020. Available: www.covidence.org

[27] Whiting PF et al . QUADAS-2: a revised tool for the quality assessment of diagnostic accuracy studies. Ann Intern Med 2011;155:529–36. 10.7326/0003-4819-155-8-201110180-00009 22007046

[28] Urbanek JK , Zipunnikov V , Harris T , et al . Validation of gait characteristics extracted from raw Accelerometry during walking against measures of physical function, mobility, fatigability, and fitness. J Gerontol A Biol Sci Med Sci 2018;73:676–81. 10.1093/gerona/glx174 28958000 PMC5905654

[29] Molina-Garcia P , Migueles JH , Cadenas-Sanchez C , et al . A systematic review on biomechanical characteristics of walking in children and adolescents with overweight/obesity: possible implications for the development of musculoskeletal disorders. Obes Rev 2019;20:1033–44. 10.1111/obr.12848 30942558

[30] Crouter SE , Schneider PL , Bassett DR . Spring-levered versus piezo-electric pedometer accuracy in overweight and obese adults. Med Sci Sports Exerc 2005;37:1673–9. 10.1249/01.mss.0000181677.36658.a8 16260966

[31] Boyer KA , Johnson RT , Banks JJ , et al . Systematic review and meta-analysis of gait mechanics in young and older adults. Exp Gerontol 2017;95:63–70. 10.1016/j.exger.2017.05.005 28499954

[32] Telfer S , Bigham JJ . The influence of population characteristics and measurement system on barefoot plantar pressures: a systematic review and meta-regression analysis. Gait Posture 2019;67:269–76. 10.1016/j.gaitpost.2018.10.030 30391749

[33] Kerrigan DC , Todd MK , Della Croce U , Croce UD . Gender differences in joint biomechanics during walking: normative study in young adults. Am J Phys Med Rehabil 1998;77:2–7. 10.1097/00002060-199801000-00002 9482373

[34] Morris ME , Huxham F , McGinley J , et al . The biomechanics and motor control of gait in Parkinson disease. Clin Biomech 2001;16:459–70. 10.1016/S0268-0033(01)00035-3 11427288

[35] Höchsmann C , Knaier R , Eymann J , et al . Validity of activity trackers, smartphones, and phone applications to measure steps in various walking conditions. Scand J Med Sci Sports 2018;28:1818–27. 10.1111/sms.13074 29460319

[36] O'Brien MW , Wojcik WR , Fowles JR . Medical-Grade physical activity monitoring for measuring step count and Moderate-to-Vigorous physical activity: validity and reliability study. JMIR Mhealth Uhealth 2018;6:e10706. 10.2196/10706 30185406 PMC6231750

[37] Chandrasekar A , Hensor EMA , Mackie SL , et al . Preliminary concurrent validity of the Fitbit-Zip and ActiGraph activity monitors for measuring steps in people with polymyalgia rheumatica. Gait Posture 2018;61:339–45. 10.1016/j.gaitpost.2018.01.035 29427859

[38] Lee JA , Williams SM , Brown DD , et al . Concurrent validation of the Actigraph gt3x+, polar active accelerometer, Omron HJ-720 and Yamax Digiwalker SW-701 pedometer step counts in lab-based and free-living settings. J Sports Sci 2015;33:991–1000. 10.1080/02640414.2014.981848 25517396

[39] Froehle AW , Nahhas RW , Sherwood RJ , et al . Age-Related changes in spatiotemporal characteristics of gait accompany ongoing lower limb linear growth in late childhood and early adolescence. Gait Posture 2013;38:14–19. 10.1016/j.gaitpost.2012.10.005 23159678 PMC3580126

[40] Grabiner PC , Biswas ST , Grabiner MD . Age-Related changes in spatial and temporal gait variables. Arch Phys Med Rehabil 2001;82:31–5. 10.1053/apmr.2001.18219 11239283

[41] Mortaza N , Abu Osman NA , Mehdikhani N . Are the spatio-temporal parameters of gait capable of distinguishing a faller from a non-faller elderly? Eur J Phys Rehabil Med 2014;50:677–91. 24831570

[42] Eshraghi A , Abu Osman NA , Karimi M , et al . Gait biomechanics of individuals with transtibial amputation: effect of suspension system. PLoS One 2014;9:e96988–e88. 10.1371/journal.pone.0096988 24865351 PMC4035274

[43] Sofuwa O , Nieuwboer A , Desloovere K , et al . Quantitative gait analysis in Parkinson's disease: comparison with a healthy control group. Arch Phys Med Rehabil 2005;86:1007–13. 10.1016/j.apmr.2004.08.012 15895349

[44] Lu M-J , Zhong W-H , Liu Y-X , et al . Sample size for assessing agreement between two methods of measurement by Bland-Altman method. Int J Biostat 2016;12. 10.1515/ijb-2015-0039. [Epub ahead of print: 01 11 2016]. 27838682

[45] Umemneku Chikere CM , Wilson K , Graziadio S , et al . Diagnostic test evaluation methodology: a systematic review of methods employed to evaluate diagnostic tests in the absence of gold standard – an update. PLoS One 2019;14:e0223832. 10.1371/journal.pone.0223832 31603953 PMC6788703

[46] Kelly P , Thomas E , Doherty A , et al . Developing a method to test the validity of 24 hour time use diaries using wearable cameras: a feasibility pilot. PLoS One 2015;10:e0142198. 10.1371/journal.pone.0142198 26633807 PMC4669185

[47] Migueles JH , Cadenas-Sanchez C , Ekelund U , et al . Accelerometer data collection and processing criteria to assess physical activity and other outcomes: a systematic review and practical considerations. Sports Med 2017;47:1821–45. 10.1007/s40279-017-0716-0 28303543 PMC6231536

[48] Ryan CG et al . The validity and reliability of a novel activity monitor as a measure of walking. Br J Sports Med 2006;40:779–84. 10.1136/bjsm.2006.027276 16825270 PMC2564393

[49] Stansfield B , Hajarnis M , Sudarshan R . Characteristics of very slow stepping in healthy adults and validity of the activPAL3™ activity monitor in detecting these steps. Med Eng Phys 2015;37:42-7. 10.1016/j.medengphy.2014.10.003 25455167

[50] Åkerberg A , Söderlund A , Lindén M . Investigation of the validity and reliability of a smartphone pedometer application. Eur J Physiother 2016;18:185–93. 10.3109/21679169.2016.1174297

[51] Ata R , Gandhi N , Rasmussen H , et al . Clinical validation of smartphone-based activity tracking in peripheral artery disease patients. npj Digital Medicine 2018;1. 10.1038/s41746-018-0073-x PMC6550212 31304343

[52] Balto JM , Kinnett-Hopkins DL , Motl RW . Accuracy and precision of smartphone applications and commercially available motion sensors in multiple sclerosis. Mult Scler J Exp Transl Clin 2016;2:205521731663475. 10.1177/2055217316634754 PMC5433404 28607720

[53] Asimina S , Chapizanis D , Karakitsios S , et al . Assessing and enhancing the utility of low-cost activity and location sensors for exposure studies. Environ Monit Assess 2018;190:155. 10.1007/s10661-018-6537-2 29464404

[54] Johnson M , Turek J , Dornfeld C , et al . Validity of the Samsung phone S health application for assessing steps and energy expenditure during walking and running: does phone placement matter? Digit Health 2016;2:205520761665274. 10.1177/2055207616652747 PMC6001243 29942556

[55] ed. Aykin N . A cross culture study on phone carrying and physical Personalization. usability and Internationalization HCI and culture. Berlin, Heidelberg: Springer Berlin Heidelberg, 2007.

[56] Alsubheen Sana’a A. , George AM , Baker A , et al . Accuracy of the vivofit activity tracker. J Med Eng Technol 2016;40:298–306. 10.1080/03091902.2016.1193238 27266422

[57] Bai Y , Hibbing P , Mantis C , et al . Comparative evaluation of heart rate-based monitors: apple Watch vs Fitbit charge HR. J Sports Sci 2018;36:1734–41. 10.1080/02640414.2017.1412235 29210326

[58] Block VJ , Zhao C , Hollenbach JA , et al . Validation of a consumer-grade activity monitor for continuous daily activity monitoring in individuals with multiple sclerosis. Mult Scler J Exp Transl Clin 2019;5:205521731988866. 10.1177/2055217319888660 PMC6876176 31803492

[59] Beltrán-Carrillo VJ , Jiménez-Loaisa A , Alarcón-López M , et al . Validity of the “Samsung Health” application to measure steps: A study with two different samsung smartphones. J Sports Sci 2019;37:788–94. 10.1080/02640414.2018.1527199 30332917

[60] YT L , Velipasalar S . Autonomous Footstep counting and traveled distance calculation by mobile devices incorporating camera and Accelerometer data. IEEE Sensors Journal 2017.

[61] Psaltos D , Chappie K , Karahanoglu FI , et al . Multimodal wearable sensors to measure gait and voice. Digit Biomark 2019;3:133–44. 10.1159/000503282 32095772 PMC7011748

[62] Kendall B , Bellovary B , Gothe NP . Validity of wearable activity monitors for tracking steps and estimating energy expenditure during a graded maximal treadmill test. J Sports Sci 2019;37:42–9. 10.1080/02640414.2018.1481723 29863968

[63] Pepa L , Verdini F , Spalazzi L . Gait parameter and event estimation using smartphones. Gait Posture 2017;57:217–23. 10.1016/j.gaitpost.2017.06.011 28667903

[64] Gaz DV , Rieck TM , Peterson NW , et al . Determining the validity and accuracy of multiple Activity-Tracking devices in controlled and Free-Walking conditions. Am J Health Promot 2018;32:1671–8. 10.1177/0890117118763273 29558811

[65] Dybus A , Paul L , Wyke S , et al . Validation of smartphone step count algorithm used in starfish smartphone application. Technol Health Care 2017;25:1157–62. 10.3233/THC-170970 28946599

[66] O’Connell S , ÓLaighin G , Quinlan LR . When a step is not a step! specificity analysis of five physical activity monitors. PLoS One 2017;12:e0169616. 10.1371/journal.pone.0169616 28085918 PMC5234787

[67] Ao B , Wang Y , Liu H , et al . Context impacts in Accelerometer-Based walk detection and step counting. Sensors 2018;18:3604. 10.3390/s18113604 PMC6263649 30352984

[68] Storm FA , Heller BW , Mazzà C . Step detection and activity recognition accuracy of seven physical activity monitors. PLoS One 2015;10:e0118723. 10.1371/journal.pone.0118723 25789630 PMC4366111

[69] Najafi B , Miller D , Jarrett BD , et al . Does footwear type impact the number of steps required to reach gait steady state?: an innovative look at the impact of foot orthoses on gait initiation. Gait Posture 2010;32:29–33. 10.1016/j.gaitpost.2010.02.016 20362453 PMC2891407

[70] Franklin S , Grey MJ , Heneghan N , et al . Barefoot vs common footwear: a systematic review of the kinematic, kinetic and muscle activity differences during walking. Gait Posture 2015;42:230–9. 10.1016/j.gaitpost.2015.05.019 26220400

[71] Svenningsen FP , de Zee M , Oliveira AS . The effect of shoe and floor characteristics on walking kinematics. Hum Mov Sci 2019;66:63–72. 10.1016/j.humov.2019.03.014 30921761

[72] Menant JC , Steele JR , Menz HB , et al . Effects of walking surfaces and footwear on temporo-spatial gait parameters in young and older people. Gait Posture 2009;29:392–7. 10.1016/j.gaitpost.2008.10.057 19041245

[73] O’Connell S , ÓLaighin G , Kelly L , et al . These shoes are made for walking: sensitivity performance evaluation of commercial activity monitors under the expected conditions and circumstances required to achieve the International daily step goal of 10,000 steps. PLoS One 2016;11:e0154956. 10.1371/journal.pone.0154956 27167121 PMC4864247

[74] Nelson MB , Kaminsky LA , Dickin DC , et al . Validity of Consumer-Based physical activity monitors for specific activity types. Med Sci Sports Exerc 2016;48:1619–28. 10.1249/MSS.0000000000000933 27015387

[75] Butte NF , Watson KB , Ridley K , et al . A youth compendium of physical activities: activity codes and metabolic intensities. Med Sci Sports Exerc 2018;50:246–56. 10.1249/MSS.0000000000001430 28938248 PMC5768467

[76] Ainsworth BE , Haskell WL , Herrmann SD , et al . 2011 compendium of physical activities: a second update of codes and Met values. Med Sci Sports Exerc 2011;43:1575–81. 10.1249/MSS.0b013e31821ece12 21681120

[77] Alinia P , Cain C , Fallahzadeh R , et al . How accurate is your activity Tracker? A comparative study of step counts in low-intensity physical activities. JMIR Mhealth Uhealth 2017;5:e106. 10.2196/mhealth.6321 28801304 PMC5572056

[78] Bunn JA , Jones C , Oliviera A , et al . Assessment of step accuracy using the consumer technology association standard. J Sports Sci 2019;37:244–8. 10.1080/02640414.2018.1491941 29958058

[79] Burton E , Hill KD , Lautenschlager NT , et al . Reliability and validity of two fitness tracker devices in the laboratory and home environment for older community-dwelling people. BMC Geriatr 2018;18:103. 10.1186/s12877-018-0793-4 29724191 PMC5934836

[80] Clay L , Webb M , Hargest C , et al . Gait quality and velocity influences activity tracker accuracy in individuals post-stroke. Top Stroke Rehabil 2019;26:412–7. 10.1080/10749357.2019.1623474 31141461

[81] Duncan MJ , Wunderlich K , Zhao Y , et al . Walk this way: validity evidence of iPhone health application step count in laboratory and free-living conditions. J Sports Sci 2018;36:1695–704. 10.1080/02640414.2017.1409855 29179653

[82] Floegel TA , Florez-Pregonero A , Hekler EB , et al . Validation of Consumer-Based hip and wrist activity monitors in older adults with varied ambulatory abilities. J Gerontol A Biol Sci Med Sci 2017;72:229–36. 10.1093/gerona/glw098 27257217 PMC6082588

[83] Huang Y , Xu J , Yu B , et al . Validity of FitBit, Jawbone up, Nike+ and other wearable devices for level and stair walking. Gait Posture 2016;48:36–41. 10.1016/j.gaitpost.2016.04.025 27477705

[84] Tam KM , Cheung SY . Validation of electronic activity monitor devices during treadmill walking. Telemed J E Health 2018;24:782–9. 10.1089/tmj.2017.0263 29364065

[85] Winter D . Kinematics. biomechanics and motor control of human movement, 2009: 45–81.

[86] Dictionaries EOL . Definition of step: Oxford Dictionaries, 2020. Available: https://en.oxforddictionaries.com/definition/step [Accessed 01 Oct 2020].

[87] Bland JM , Altman DG . Statistical methods for assessing agreement between two methods of clinical measurement. Lancet 1986;1:307–10. 2868172

[88] Vet H . Observer reliability and agreement. Wiley StatsRef: Statistics Reference Online, 2014.

[89] Zaki R , Bulgiba A , Ismail R , et al . Statistical methods used to test for agreement of medical instruments measuring continuous variables in method comparison studies: a systematic review. PLoS One 2012;7:e37908–e08. 10.1371/journal.pone.0037908 22662248 PMC3360667

[90] Ludbrook J . Statistical techniques for comparing measurers and methods of measurement: a critical review. Clin Exp Pharmacol Physiol 2002;29:527–36. 10.1046/j.1440-1681.2002.03686.x 12060093

[91] MAPE . Encyclopedia of production and manufacturing management. Boston, MA: Springer US, 2000: 462–62.

[92] Validation of temporal gait metrics from three IMU locations to the gold standard force plate. 2016 38th annual International Conference of the IEEE engineering in medicine and biology Society (EmbC) 2016:16–20. 10.1109/EMBC.2016.7590790 28268416

[93] Ngueleu AM , Blanchette AK , Maltais D , et al . Validity of instrumented Insoles for step counting, posture and activity recognition: a systematic review. Sensors 2019;19:2438. 10.3390/s19112438 PMC6603748 31141973

