# Supplementary material for: Recommendations for determining the validity of consumer wearable and smartphone step count: expert statement and checklist of the INTERLIVE network
Source: Br J Sports Med. 2020 Dec 24;55(14):780–93. doi: 10.1136/bjsports-2020-103147 (PMC8273687; doi:10.1136/bjsports-2020-103147)
Supplement: Supplementary data [file bjsports-2020-103147supp001.pdf]

Online Supplementary Data

Appendix 1. Search terms used in Embase, PubMed, and Web of Science databases.

Embase	Web of Science	Pubmed
**Wearables and smartphone**	**Wearables and smartphone**	**Wearables and smartphone**
('wearable electronic devices'/exp **OR** 'wearable electronic devices' **OR** wearable* **OR** smartwatch * **OR** (('smart'/exp **OR** 'smart') AND watch*) **OR** (('smart'/exp **OR** smart) AND band*) **OR** (('smart'/exp **OR** smart) AND bracelet*) **OR** 'smartphone'/exp **OR** 'smartphone' **OR** smartphone*)	ALL FIELDS: (wearable* **OR** smartwatch* **OR** "smart watch" **OR** "smart watches" **OR** smartphone* **OR** (smart AND band*) **OR** (smart AND bracelet*))	("Wearable Electronic Devices"[Mesh] **OR** wearable* **OR** smartwatch* **OR** (smart AND watch*) **OR** (smart AND band*) **OR** (smart AND bracelet*) **OR** "Smartphone"[Mesh] **OR** smartphone*)
**Outcome**	**Outcome**	**Outcome**
**AND** (step*)	**AND** (step*)	**AND** (step*)
**Study design**	**Study design**	**Study design**
**AND** ('reproducibility of results'/exp **OR** 'reproducibility of results' **OR** 'validity'/exp **OR** 'validity' **OR** 'validation'/exp **OR** 'validation' **OR** validate **OR** 'compa rison'/exp **OR** 'comparison' **OR** 'reliability'/exp **OR** 'reliability' **OR** reliable)	**AND** (validity **OR** validation **OR** validate **OR** comparison **OR** reliability **OR** reliable)	**AND** ("Reproducibility of Results"[Mesh] **OR** validity **OR** validation **OR** validate **OR** comparison **OR** reliability **OR** reliable)

Appendix 2. QUADAS-2 methodology, criteria for the risk of bias assessment, and the percentage of studies meeting these criteria.

As advised by the QUADAS-2 guidelines, a series of signalling questions were developed by the consortium members. The risk of bias assessment was conducted by three authors whereby each author independently rated two-thirds of the papers using a cross-over methodology to ensure each paper was reviewed by two authors. Any discrepancies were resolved by arbitration with the third author. Once a consensus was agreed upon, each study was given a risk of bias rating (high, low, or unclear) for each of the four headings (patient selection, index measure, criterion measure, and study flow and timing). If all signalling questions for a domain were answered "yes" then risk of bias was deemed to be "low". If any signalling question was answered "no", then risk of bias was deemed to be 'high'. The "unclear" category was only used when insufficient data were reported to permit a judgment[1].

Criteria items	N studies meeting criterion		
	Laboratory (N=57)	Semi-free (N=9)	Free-living (N=30)
**Domain 1:** Patient Selection			
1. Did the study avoid inappropriate exclusions?	42 (74%)	9 (100%)	24 (80%)
**Domain 2:** Index measure			
2. Was the wearable/ smartphone used in its ecological context?	47 (82%)	8 (89%)	28 (93%)
**Domain 3:** Criterion measure			
3. Is the reference standard a 'Gold standard'?	29 (51%)	4 (44%)	1 (3%)
**Domain 4:** Flow and timing			
4. Did they provide adequate information about data synchronization?	16 (28%)	3 (33%)	8 (27%)
5. Did all participants receive the same reference standard?	53 (93%)	9 (100%)	29 (97%)
6. Were all participants included in the analysis or appropiate exclusion reasons were provided?	45 (79%)	9 (100%)	21 (70%)

Appendix 3. Summary of the validation methodologies used in the laboratory-condition studies (N = 57).

Nº	Author	Population N (age ± SD or range; % girls)	Testing Protocol	Criterion Measure	Index Measure; Placement	Data Synchro.	Statistics
1	Åkerberg et al. 2016 [2]	Healthy adults 20 (30-61; 55%)	Walking in sloping and flat surfaces, and climbing stairs	Visual observation	Smartphone; Special vest to hold the phone	Data collected simultaneously	Hypo. and relative error
2	Alsubheen et al. 2016[3]	Healthy adults 13 (40.0 ± 11.9; 38%)	Treadmill walking	Video	Activity tracker; Wrist	Data collected simultaneously	Hypo.
3	An et al. 2017[4]	Healthy adults 35 (31.0 ± 11.8; 51%)	Treadmill walking and running	Visual observation	Activity trackers; Arm, wrist or waist	Data collected simultaneously	B&A, equivelance test, corr. and MAPE
4	Arch et al. 2017[5]	Unilateral transtibial amputation 50 (58.1 ± 10.5; 28%)	Overground walking tests	Visual observation	Activity tracker; Attached to prosthesis	ND	RR, corr., regr. and APE
5	Ata et al 2018[6]	Peripheral Artery Disease 182 (69.5 ± 13.1; 23%)	6 minute walk test	Visual observation	Smartphone; Hand, pocket and purse/bag	ND	B&A and regr.
6	Balmain et al 2019[7]	Healthy adults 36 (21.0 ± 1.0; 53%)	Treadmill and overground walking	Video	Smartphone & smart clothing; Right hip and feet	ND	B&A and regr.
7	Balto et al 2016[8]	Multiple sclerosis 45 (47.7 ± 10.0; ND)	Treadmill walking	Visual observation with ≥2 observers	Smartphones & activity trackers; Pocket and wrist	ND	Corr. and MPE
8	Beltrán-Carrillo et al 2019[9]	Healthy adults 16 (28.8 ± 8.9; 50%)	Overground walking on straight track	Video	Smartphones; Waist, arm and hand	Wear time and/or task time considered	B&A, hypo., corr., RMSE and RB
9	Block et al 2019[10]	Multiple Sclerosis 61 (50.0 ± 14.2; 72%)	2 min walk test	Visual observation	Activity trackers; Non-dominant wrist	ND	B&A, hypo., RR and regr.
10	Brodie et al 2018[11]	Healthy adults 48 (28.8 ± 8.9; 58%)	Overground walking	Visual observation	Smartphone; Phone fixed at the posterior hip	ND	Hypo. and APE
11	Buckinx et al 2017[12]	Healthy adults 24 (46.3 ± 3.6; 50%)	Treadmill walking	Video	Activity trackers; Right ankle and right hip	ND	RR
12	Bunn et al 2019[13]	Healthy adults 24 (26.5 ± 11.5; 50%)	Treadmill walking	Video with ≥2 observers	Activity trackers; Manufacturers' guidance	Data collected simultaneously	Hypo., Equiv., MPE
13	Burton et al 2018[14]	Healthy adults 31 (74.2 ± 5.8; 65%)	Overground walking	Video with ≥2 observers	Activity trackers; Wrist	ND	Hypo. and RR

14	Chandrasekar et al 2018[15]	Polymyalgia rheumatica 31 (69.2 ± 8.8; 89%)	Overground walking	Video with ≥2 observers	Activity trackers; Right hip and midline of the shirt	Wear time and/or task time considered	B&A, corr. and MAPE
15	Clay et al 2019[16]	Stroke patients 21 (65.6 ± 8.2; 58%)	Overground walking	Video with ≥2 observers	Activity tracker; Waist band of non-paretic side	ND	B&A, RR, corr. and regr.
16	De Ridder et al 2019[17]	Crutch walking 30 (24.9 ± 5.3; 50%)	Overground walking	Visual observation with ≥2 observers	Activity trackers; Both wrists and waist	ND	B&A, hypo., RR and % bias
17	Duncan et al 2018[18]	Healthy adults 33 (25.9 ± 9.4; 67%)	Treadmill walking	Video with ≥2 observers	Smartphones; ND	Wear time and/or task time considered	B&A, hypo., RR and regr.
18	Ebara et al 2017[19]	Healthy adults 5 (31.2 ± 8.5; 0%)	Overground walking	Visual observation	Smartphones; Bust strap around chest	ND	B&A, hypo., RR and corr.
19	Floegel et al 2017[20]	Adults with different fitness level 99 (78.9 ± 8.6; 71%)	Overground walking	Video with ≥2 observers	Activity trackers; Non-dominant hip and wrist	Wear time and/or task time considered	B&A, Equiv., RR, MAPE and MPE
20	Fokkema et al 2017[21]	Healthy adults 31 (32.0 ± 12.0; 48%)	Treadmill walking	Video	Activity trackers and smartphone; Wrist, hip and pocket	Data collected simultaneously	B&A, RR and regr.
21	Gaz et al 2018[22]	Healthy adults 32 (35.8 ± 7.8; 48%)	Treadmill walking	Visual observation with ≥2 observers	Activity trackers; Dominant wrist and hip	Data collected simultaneously	GLMM
22	Hernández-Belmonte et al 2019[23]	Healthy adults 10 (ND; 0%)	Overground walking, jogging and running	Video	Activity tracker; Upper back	Data collected simultaneously	B&A, RR and corr.
23	Höchsmann et al 2018 [24]	Healthy adults 20 (18-70; 70%)	Treadmill and overground walking and running with different inclinations and stairs	Video	Activity trackers and smartphones; Non-dominant wrist, pocket and strapped in the arm	Data collected simultaneously	MAPE
24	Huang et al 2016[25]	Healthy adults 40 (23.6 ± 2.1; 25%)	Treadmill and overground walking and stair test	Video with ≥2 observers	Activity trackers; Both wrists	Data collected simultaneously	B&A, hypo. and MAPE
25	Hurt et al 2018[26]	Healthy adults 57 (28.3 ± 9.9; 46%)	Overground walking	Visual observation	Smartphones; Frontal pocket	Data collected simultaneously	Corr. and GLMM
26	Johnson et al 2016[27]	Healthy adults 29 (21.7 ± 1.6; 52%)	Overground walking	Research grade wearable device	Smartphones; Held in the hand and right pocket	Task time considered	B&A, hypo. and corr.

27	Jones et al 2018[28]	Healthy adults 30 (33.0 ± 8.0; 60%)	Treadmill jogging and running	Video	Activity trackers; Both wrists	Data collected simultaneously	RR, MAPE and standard error
28	Kendall et al 2019[29]	Healthy adults 50 (25.8 ± 8.1; 50%)	Maximal treadmill test	Visual observation	Activity trackers; Both wrists and right hip	Data collected simultaneously	Hypo. and RR
29	Lamont et al 2018[30]	Mild-moderate Parkison's Disease 33 (69.0 ± 8.1; 50%)	Walking in different surfaces	Research grade wearable device	Activity trackers; Both wrists	Data collected simultaneously	B&A, hypo., RR and MAPE
30	Lebleu et al 2020[31]	Healthy adults 60 (23.4 ± 1.3; 48%)	Overground walking circuit	Research grade wearable device	Smartphone; Both wrists and non-dominant hip	Data collected simultaneously	B&A, RR and MAPE
31	Leong et al 2017[32]	Healthy adults 48 (19-25; 73%)	Treadmill walking	Visual observation	Smartphone; Right pocket	Data collected simultaneously	RR, corr. and MAPE
32	Liew et al 2020[33]	Healthy adults 24 (23-30; 50%)	Overground walking	Visual observation	Activity tracker; Wrist	Data collected simultaneously	RR, corr. and MAPE
33	Lu et al 2017[34]	ND	Overground walking in different directions	Visual observation	Smartphone; Waist holder, pocket, backpack or hands	Data collected simultaneously	MAPE
34	Magistro et al 2018[35]	Healthy older adults 60 (75.0 ± 7.0; 50%)	Overground walking and stairs test	Video	Activity trackers; Both wrists	Data collected simultaneously	B&A, RR and APE
35	Major et al 2016[36]	Healthy adults 20 (28.0 ± 5.0; 50%)	Overground walking	Video	Smartphone; Right pocket	Data collected simultaneously	B&A, RR and corr.
36	Massouh et al 2019[37]	Cesarean delivery patients 48 (32.0 ± 6.0; 100%)	Overground walking	Visual observation	Activity trackers; Non-dominant wrist	Data collected simultaneously	B&A, RR and corr.
37	Montes et al 2018[38]	ND 49 (23.4 ± 6.7; 48%)	Treadmill walking	Visual observation	Smart shirt; Worn as normal	Data collected simultaneously	RR and corr.
38	Montoye et al 2017[39]	Healthy adults 32 (23.5 ± 1.3; 44%)	Treadmill walking and running, lying, standing, sitting and cycling	Research grade wearable device	Smart shirt and activity tracker; Shirt worn as normal and tracker on non-dominant wrist	Synchro. issues mentioned but not discussed	B&A, hypo., corr. and MAPE
39	Munck et al 2018[40]	Healthy adults 22 (27.0 ± 7.3; 50%)	Treadmill walking	ND	Activity trackers; Wrist	ND	MPE
40	Orr et al 2015[41]	ND 29 (27.1 ± 8.3; ND)	Overground and treadmill walking	Video and participant counted their own steps	Smartphones; Held in participants' hands	ND	Hypo.

41	Pepa et al 2017[42]	Healthy adults 22 (22-30; 27%)	Overground walking	Research grade wearable device	Smartphone; Lateral side of the hip and posterior pelvis	Jump used as synchro.	Hypo. and corr.
42	Polese et al 2019[43]	Stroke patients 37 (62.0 ± 11.0; 24%)	Overground walking	Video	Smartphone; Front pockets of the participants' paretic leg	ND	Corr.
43	Presset et al 2018[44]	ND 37 (30-60; 35%)	Treadmill walking	Research grade wearable device	Smartphone; Attached to the belt, the biceps and a jacket	ND	B&A
44	Psaltos et al 2019[45]	Healthy adults 40 (34.8 ± 10.2; 53%)	Overground walking	Research grade wearable device	Activity trackers and smartphones; Trackers: wrist, phones: attached to 4th lumbar vertebrae	ND	B&A and corr.
45	Rüdiger et al 2019[46]	Healthy adults 32 (74.8 ± 5.9; 56%)	Overground walking	Visual observation and research grade wearable device	Activity tracker; Non-dominant arm	ND	B&A and corr.
46	Schaffer et al 2017[47]	Stroke patients 24 (54.0 ± 13.4; 42%)	Overground walking	Video	Activity trackers; Wrists of both paretic and non-paretic arms	ND	B&A and hypo.
47	Schmal et al 2018[48]	Post-operative patients 22 (81.0 ± 8.0; 50%)	ND	Video	Activity trackers; Wrist and ankle	ND	Corr.
48	Smith et al 2019[49]	Lower-limb prosthesis users 32 (49.7 ± 14.0; 34%)	Overground walking	Visual observation	Activity trackers; Both wrists	Wear and task time considered	Hypo.
49	Tam et al 2018[50]	Healthy adults 30 (32.1 ± 8.7; 50%)	Treadmill walking	Video with ≥2 observers	Activity trackers; Non-dominant wrist	ND	Corr.
50	Tedesco et al 2019[51]	Healthy older people 18 (69.0 ± 3.2; 61%)	Treadmill walking	Video	Activity trackers; Both wrists	Synchro. issues mentioned but not discussed	MAPE, MPE, RMSE, AME, MAD
51	Thorup et al 2017[52]	Healthy adults and cardiac patients 44 (53.0 ± 7.4; 27%)	Treadmill walking	Research grade wearable device	Activity tracker; Elastic belts, two at the heart level and two at the waist	ND	RB
52	Tophøj et al 2018[53]	Healthy adults 20 (25.6 ± 2.0; 50%)	Treadmill walking	Visual observation	Activity trackers; Non-dominant wrist	ND	MAPE and MAD

53	Van Oeveren et al 2018[54]	Healthy adults 22 (28.0 ± 2.9; 41%)	Overground walking	Video	Smartphones; Pocket, strapped to the arm and the back waist	Jump used as synchro.	Hypo.
54	Veerabhadrappa et al 2018[55]	Healthy adults 71 (18-55; 34%)	Treadmill walking	Video	Activity tracker; Left wrist	Wear and task time considered	Corr.
55	Wahl et al 2017[56]	Healthy adults 20 (26.1 ± 2.8; 50%)	Treadmill and overground walking	Research grade wearable device	Activity trackers; Armband and backside of the pelvis. Other trackers ND	ND	B&A, RR, MAPE and TE
56	Wong et al 2018[57]	Healthy adults 25 (25.0 ± 6.7; 48%)	Treadmill walking	Video	Activity tracker; Right hip	Wear and task time considered	Hypo.
57	Xie et al 2018[58]	Healthy adults 44 (22.2 ± 2.2; 48%)	Overground walking and running	Video	Activity trackers and smartphones; Both wrists and pocket	Synchro. issues mentioned but not discussed	Corr. and MAPE

Abbreviations. Synchro.: synchronization; ND: Not disclosed; SD: standard deviation.

Statistics code. B&A: Bland & Altman; Hypo.: hypothesis test; Equiv.: equivalence test; RR: relative reliability; Corr.: correlation; Regr.: regression; MAPE: mean absolute percentage error; APE: absolute percentage error; MPE: mean percentage error; RMSE: root-mean-square deviation; RB: relative bias; AME: absolute mean error; %bias: percentage of bias; GLMM: generalized linear mixed model; MAD: median absolute difference; SEM: standard error of measurement; TE: typical error.

Appendix 4. Summary of the validation methodologies used in the semi-free-living studies (N = 9).

Nº	Author	Population N (age ± SD or range; % girls)	Testing Protocol	Criterion Measure	Index Measure; placement	Data Synchro.	Statistics
1	Bai et al 2018[59]	Healthy adults 41 (32.0 ± 11.0; 38%)	Sedentary activities, aerobic exercise and household activities	Research grade wearable device	Activity trackers; Left wrist	ND	B&A, Equiv., corr., MAPE, MPE and RMSE and
2	Bort-Roig et al 2018[60]	Healthy adults 17 (26.0 ± 3.0; 59%)	Overground walking, stairs and work simulation	Research grade wearable device	Smartphone; Pouch in the mid-to-front point of the thigh	ND	RR and AME
3	Genovese et al 2017[61]	Healthy adults 8 (38.5 ± 11.8; 38%)	Sedentary activities, ambulatory and household activities	Visual observation	Activity trackers; Non-dominant wrist and waist	Data collected simultaneously	RMSE and AME
4	Imboden et al 2018[62]	Healthy adults 30 (69.5 ± 13.1; 23%)	Sedentary, household and ambulatory activities	Visual observation	Activity trackers; Left hip and non-dominant wrist	Data collected simultaneously	B&A, hypo., corr. and MAPE
5	Nelson et al 2016[63]	Healthy adults 30 (48.9 ± 19.4; 50%)	Sedentary, household and ambulatory activities	Visual observation	Activity trackers; Left hip and non-dominant wrist	Data collected simultaneously	Hypo., MAPE, RMSE and MAE
6	O'Connell et al 2017[64]	Healthy adults 37 (39.0 ± 13.9; 68%)	Work simulation, vehicles, household and fitness activities	Video	Activity trackers; Both hips, right wrist and chest	Wear and task time considered	Regr.
7	Tedesco et al 2019[65]	Healthy older adults 18 (69.3 ± 2.8; 61%)	Daily life activities	Video	Activity trackers; Both wrists	Synchro. issues mentioned but not discussed	MAPE, MPE, RMSE, AME and MAD
8	Ummels et al 2018[66]	Several diseases 130 (61.5 ± 11.1; 58%)	Daily life activities	Video	Activity trackers and smartphones; Pocket and wrist	ND	B&A, 2 and 5
9	Wendel et al 2018[67]	Parkinson's disease 33 (65.5 ± 9.4; 42%)	Daily life activities	Video	Activity trackers; Left wrist and left hip	ND	B&A, 4, 7

Abbreviations. Synchro.: synchronization; ND: Not disclosed; SD: standard deviation.

Statistics code. B&A: Bland & Altman; Hypo.: hypothesis test; Equiv.: equivalence test; RR: relative reliability; Corr.: correlation; Regr.: regression; MAPE: mean absolute percentage error; APE: absolute percentage error; MPE: mean percentage error; RMSE: root-mean-square deviation; RB: relative bias; AME: absolute mean error; %bias: percentage of bias; GLMM: generalized linear mixed model; MAD: median absolute difference; SEM: standard error of measurement; TE: typical error.

Appendix 5. Summary of the validation methodologies used in the free-living studies (N = 30).

Nº	Author	Population N (age ± SD or range; % girls)	Testing Protocol	Criterion Measure	Index Measure; placement	Data Synchro.	Statistics
1	Amagasa et al 2019[68]	Healthy adults 54 (31.0 ± 10.0; 52%)	At home	Research grade wearable device	Smartphone; Carried as usual	Data collected simultaneously	B&A, hypo., RR, corr. and regr.
2	An et al 2017[4]	Healthy adults 35 (31.0 ± 11.8; 51%)	24h of free-living	Research grade wearable device	Activity trackers; Upper arm, wrist and waist	Data collected simultaneously	B&A, equiv., corr. and MAPE
3	Arch et al 2018[5]	Unilateral transtibial amputation 50 (58.1 ± 10.5; 28%)	7 days of free-living	Research grade wearable device	Activity tracker; Attached to the prosthesis	ND	RR, corr., regr. and APE
4	Block et al 2019[10]	Multiple Sclerosis 61 (54.0 ± 11.4; 72%)	7 days of free-living	Research grade wearable device	Activity trackers; Non-dominant wrist	Wear time considered	B&A, hypo., RR and regr.
5	Bort-Roig et al 2018[60]	Healthy adults 17 (26.0 ± 3.0; 59%)	2h of free-living	Research grade wearable device	Smartphone; Pouch in the mid-to-front point of the thigh	ND	RR and B&Aequiv.
6	Burton et al 2018[14]	Healthy adults 31 (74.2 ± 5.8; 65%)	14 days of free-living	Research grade wearable device	Activity tracker; Wrist	ND	Hypo. and RR
7	Chu et al 2017[69]	Healthy adults 107 (26-42; 66%)	At least 4 days of free-living	Research grade wearable device	Activity tracker; Non-dominant wrist	Synchro. issues mentioned but not discussed	B&A, hypo., RR, corr. and MAPE
8	Collins et al 2019[70]	Knee osteoarthritis patients 15 (68.0 ± 8.0; 67%)	7 days of free-living	Research grade wearable device	Activity tracker; Non-dominant wrist	ND	RR and B&ARR
9	Degroote et al 2018[71]	Healthy adults 36 (39.4 ± 17.8; 50%)	2 days of free-living	Research grade wearable device	Activity trackers; Non-dominant wrist	Wear time considered	B&A, RR and corr.
10	Dominick et al 2016[72]	Healthy adults 19 (19-37; 79%)	14 days of free-living	Research grade wearable device	Activity tracker; Dominant wrist	Wear time considered	Hypo., corr. and B&Acorr.
11	Douma et al 2018[73]	Cancer patients 89 (63.0 ± 11.5; 38%)	7 days of free-living	Research grade wearable device	Smartphone; Pocket or attached to a belt	Wear time considered	B&A, RR and regr.
12	Duncan et al 2018[18]	Healthy adults 33 (25.9 ± 9.4; 67%)	7 days of free-living	Research grade wearable device	Smartphone; ND	Wear time considered	B&A, hypo., RR and regr.

13	Ferguson et al 2015[74]	Healthy adults 21 (32.8 ± 10.2; 52%)	2 days of free-living	Research grade wearable device	Activity trackers; Left wrist and right hip	Wear time considered	B&A, corr. and B&Aregr.
14	Gill et al 2018[75]	Healthy adults 21 (30-65; 0%)	7 days of free-living	Research grade wearable device	Activity tracker; Pocket	Wear time considered	B&A, corr. and B&Aequiv.
15	Gomersall et al 2016[76]	Healthy adults 32 (39.6 ± 11.0; 90%)	7 days of free-living	Research grade wearable device	Activity trackers; Belt, pocket and both wrists	Data collected simultaneously	B&A and corr.
16	Höchsmann et al 2020[77]	Healthy adults 30 (23-32; 62%)	3 days of free-living	Research grade wearable device	Activity trackers and smartphones; Non-dominant wrist, both hips and pocket	Data collected simultaneously	B&A, RR and MAPE
17	Hartwig et al 2019[78]	Healthy children and adolescents 592 (13.5 ± 0.5; 49%)	Physical education classes	Research grade wearable device	Activity tracker; Hip	Data collected simultaneously	B&A, corr. and regr.
18	Lebleu et al 2020[31]	Healthy adults 60 (39.4 ± 12.0; 0%)	24h of free-living	Research grade wearable device	Smartphones; Both wrists and hip on non-dominant side	Data collected simultaneously	B&A, RR and MAPE
19	Leong et al 2017[32]	Healthy adults 48 (19-25; 73%)	7 days of free-living	Research grade wearable device	Smartphone; Pocket, right tight and left arm	Data collected simultaneously	RR, corr. and MAPE
20	Liew et al 2020[33]	Healthy adults 40 (23-30; 50%)	At least 4 days of free-living	Research grade wearable device	Activity tracker; Wrist	Data collected simultaneously	RR, corr. and B&Aregr.
21	Middelweerd et al 2017[79]	Healthy adults 34 (23.9 ± 3.9; 68%)	7 days of free-living	Research grade wearable device	Activity tracker; Right hip with a waist belt	Data collected simultaneously and wear time considered	B&A, RR, APE and B&Acorr.
22	Mooses et al 2018[80]	Healthy children 147 (9-10; 50%)	School ground	Research grade wearable device	Activity tracker; Hip	Data collected simultaneously	B&A, hypo. and corr.
23	Orr et al 2015[41]	ND 29 (27.1 ± 8.3; ND)	3 days of free-living	Research grade wearable device	Smartphones; Held in participant hands	Wear time considered	Hypo.
24	Rosenberger et al 2016[81]	ND 40 (21-876; 53%)	24h of free-living	Research grade wearable device	Activity trackers; Right wrist	Wear time considered	MAPE
25	Rozanski et al 2018[82]	Stroke patients 37 64.4 ± 15.9; 53%)	2 separated days of free-living	Research grade wearable device	Activity trackers; Wrist	ND	Hypo. and corr.

26	Stamatelopoulou et al 2018[83]	ND<br>21 (ND;ND)	7 days of free-living	Research grade wearable device	Activity tracker and smartphone; Wrist and pocket or bag	ND	Corr.
27	Tedesco et al 2019[65]	Healthy older adults<br>20 (70.6 ± 3.0; 55%)	1 days of free-living	Research grade wearable device	Activity trackers; Non-dominant wrist	ND	RR
28	Toth et al 2018[84]	Healthy adults<br>12 (35.0 ± 13.0; 50%)	24h of free-living	Video with ≥2 observers	Activity trackers; Random wrist and left hip	Wear time considered	Hypo. and MAPE
29	Voss et al 2017[85]	Congenital heart disease<br>40 (13.0 ± 2.2; 53%)	7 days of free-living	Research grade wearable device	Activity tracker; Manufactured guidelines	Wear time considered	B&A and RR
30	Yang et al 2019[86]	Healthy adults<br>120 (13.0 ± 2.5; 52%)	7 days of free-living	Research grade wearable device	Activity tracker; Non-dominant wrist	ND	B&A, RR and corr.

Abbreviations. Synchro.: synchronization; ND: Not disclosed; SD: standard deviation.

Statistics code. B&A: Bland & Altman; Hypo.: hypothesis test; Equiv.: equivalence test; RR: relative reliability; Corr.: correlation; Regr.: regression; MAPE: mean absolute percentage error; APE: absolute percentage error; MPE: mean percentage error; RMSE: root-mean-square deviation; RB: relative bias; AME: absolute mean error; %bias: percentage of bias; GLMM: generalized linear mixed model; MAD: median absolute difference; SEM: standard error of measurement; TE: typical error.

Appendix 6. QUADAS-2 risk of bias for the laboratory based studies.

Article Number	Author	Patient Selection	Index measure	Criterion measure	Flow & Timing
1	Åkerberg et al. 2016 [2]	Low	Low	High	Low
2	Alsubheen et al. 2016[3]	Low	Low	Low	High
3	An et al. 2017[4]	Low	Low	High	Low
4	Arch et al. 2017[5]	High	High	High	High
5	Ata et al 2018[6]	Low	Low	High	Low
6	Balmain et al 2019[7]	Low	Low	Low	High
7	Balto et al 2016[8]	Low	Low	High	High
8	Beltrán-Carrillo et al 2019[9]	Low	High	Low	High
9	Block et al 2019[10]	Low	Low	High	High
10	Brodie et al 2018[11]	Low	High	High	High
11	Buckinx et al 2017[12]	Low	Low	Low	High
12	Bunn et al 2019[13]	Low	Low	Low	High
13	Burton et al 2018[14]	Low	Low	Low	High
14	Chandrasekar et al 2018[15]	Low	Low	Low	Low
15	Clay et al 2019[16]	Low	Low	Low	High
16	De Ridder et al 2019[17]	High	Low	High	High
17	Duncan et al 2018[18]	Low	Low	Low	High
18	Ebara et al 2017[19]	High	High	High	High
19	Floegel et al 2017[20]	Low	Low	Low	High
20	Fokkema et al 2017[21]	Unclear	Low	Unclear	High
21	Gaz et al 2018[22]	Low	Low	High	Low
22	Hernández-Belmonte et al 2019[23]	Low	Low	Low	High
23	Höchsmann et al 2018 [24]	Low	Low	Low	High
24	Huang et al 2016[25]	Low	Low	Low	High
25	Hurt et al 2018[26]	Low	Low	High	High
26	Johnson et al 2016[27]	Low	Low	High	Low
27	Jones et al 2018[28]	Low	Low	Low	High
28	Kendall et al 2019[29]	Unclear	High	High	High
29	Lamont et al 2018[30]	Low	Low	High	High
30	Lebleu et al 2020[31]	Low	Low	High	High
31	Leong et al 2017[32]	Low	Low	High	High
32	Liew et al 2020[33]	Low	Low	High	High
33	Lu et al 2017[34]	High	High	High	High

34	Magistro et al 2018[35]	Low	Low	Low	Low
35	Major et al 2016[36]	Low	Low	Low	Low
36	Massouh et al 2019[37]	Low	Low	High	High
37	Montes et al 2018[38]	Unclear	Low	High	High
38	Montoye et al 2017[39]	Low	Low	High	High
39	Munck et al 2018[40]	Low	Low	High	High
40	Orr et al 2015[41]	Unclear	Low	Low	Low
41	Pepa et al 2017[42]	Unclear	High	Low	High
42	Polese et al 2019[43]	Low	Low	Low	High
43	Presset et al 2018[44]	Unclear	Low	Low	High
44	Psaltos et al 2019[45]	Unclear	High	Low	High
45	Rüdiger et al 2019[46]	Low	Low	High	High
46	Schaffer et al 2017[47]	Low	Low	Low	High
47	Schmal et al 2018[48]	Low	Low	Low	High
48	Smith et al 2019[49]	Low	Low	High	High
49	Tam et al 2018[50]	Low	Low	Low	High
50	Tedesco et al 2019[51]	Low	Low	Low	Low
51	Thorup et al 2017[52]	Low	Low	High	High
52	Tophøj et al 2018[53]	Unclear	Low	High	High
53	Van Oeveren et al 2018[54]	Unclear	Low	Low	Low
54	Veerabhadrappa et al 2018[55]	Low	Low	Low	Low
55	Wahl et al 2017[56]	Unclear	High	Low	High
56	Wong et al 2018[57]	Low	Low	Low	Low
57	Xie et al 2018[58]	Low	Low	Low	High
	High/unclear Risk of Bias Count	14	9	26	44
	% High Risk of Bias	25%	16%	46%	77%

Appendix 7. QUADAS-2 risk of bias for the semi-free-living studies.

Article Number	Author	Patient Selection	Index measure	Criterion measure	Flow & Timing
1	Bai et al 2018[59]	Low	Low	High	High
2	Bort-Roig et al 2018[60]	Low	High	High	High
3	Genovese et al 2017[61]	Low	Low	High	High
4	Imboden et al 2018[62]	Low	Low	High	High
5	Nelson et al 2016[63]	Low	Low	High	Low
6	O'Connell et al 2017[87]	Low	Low	Low	Low
7	Tedesco et al 2019[65]	Low	Low	Low	Low
8	Ummels et al 2018[66]	Low	Low	Low	High
9	Wendel et al 2018[67]	Low	Low	Low	High
	High/unclear Risk of Bias Count	0	1	5	6
	% High Risk of Bias	0%	11%	56%	67%

Appendix 8. QUADAS-2 risk of bias for the free-living studies.

Article Number	Author	Patient Selection	Index measure	Criterion measure	Flow & Timing
1	Amagasa et al 2019[68]	Low	Low	High	High
2	An et al 2017[4]	Low	Low	High	High
3	Arch et al 2018[5]	High	High	High	High
4	Block et al 2019[10]	High	Low	High	High
5	Bort-Roig et al 2018[60]	Low	High	High	High
6	Burton et al 2018[14]	Low	Low	High	High
7	Chu et al 2017[69]	Low	Low	High	High
8	Collins et al 2019[70]	Low	Low	High	High
9	Degroote et al 2018[71]	Low	Low	High	High
10	Dominick et al 2016[72]	Low	Low	High	Low
11	Douma et al 2018[73]	Low	Low	High	High
12	Duncan et al 2018[18]	Low	Low	High	High
13	Ferguson et al 2015[74]	Low	Low	High	High
14	Gill et al 2018[75]	Low	Low	High	Low
15	Gomersall et al 2016[76]	Low	Low	High	High
16	Höchsmann et al 2020[77]	Low	Low	High	High
17	Hartwig et al 2019[78]	Unclear	Low	High	High
18	Lebleu et al 2020[31]	Low	Low	High	High
19	Leong et al 2017[32]	Low	Low	High	High
20	Liew et al 2020[33]	Low	Low	High	High
21	Middelweerd et al 2017[79]	Low	Low	High	Low
22	Mooses et al 2018[80]	High	High	High	Low
23	Orr et al 2015[41]	Low	Low	High	Low
24	Rosenberger et al 2016[81]	Unclear	Low	High	High
25	Rozanski et al 2018[82]	Low	Low	High	High
26	Stamatelopoulou et al 2018[83]	Unclear	Low	High	High
27	Tedesco et al 2019[65]	Low	Low	High	High
28	Toth et al 2018[84]	Low	Low	Low	Low
29	Voss et al 2017[85]	Low	Low	High	Low
30	Yang et al 2019[86]	Low	Low	High	High
	High Risk of Bias Count	6	3	29	23
	% High Risk of Bias	20%	10%	97%	77%

Supplemental Data Reference List

1. Whiting PF, Rutjes AW, Westwood ME, et al. QUADAS-2: a revised tool for the quality assessment of diagnostic accuracy studies. *Annals of internal medicine* 2011;155(8):529-36. doi: 10.7326/0003-4819-155-8-201110180-00009 [published Online First: 2011/10/19]
2. Åkerberg A, Söderlund A, Lindén M. Investigation of the validity and reliability of a smartphone pedometer application. *European Journal of Physiotherapy* 2016;18(3):185-93. doi: 10.3109/21679169.2016.1174297
3. Alsubheen SA, George AM, Baker A, et al. Accuracy of the vivofit activity tracker. *Journal of medical engineering & technology* 2016;40(6):298-306. doi: 10.1080/03091902.2016.1193238
4. An HS, Jones GC, Kang SK, et al. How valid are wearable physical activity trackers for measuring steps? *European journal of sport science* 2017;17(3):360-68. doi: 10.1080/17461391.2016.1255261
5. Arch ES, Sions JM, Horne J, et al. Step count accuracy of StepWatch and FitBit One™ among individuals with a unilateral transtibial amputation. *Prosthetics and orthotics international* 2018;42(5):518-26. doi: 10.1177/0309364618767138
6. Ata R, Gandhi N, Rasmussen H, et al. Clinical validation of smartphone-based activity tracking in peripheral artery disease patients. *npj Digital Medicine* 2018;1(1) doi: 10.1038/s41746-018-0073-x
7. Balmain BN, Tuttle N, Bailey J, et al. Using Smart Socks to Detect Step-count at Slow Walking Speeds in Healthy Adults. *International journal of sports medicine* 2019;40(2):133-38. doi: 10.1055/a-0732-5621
8. Balto JM, Kinnett-Hopkins DL, Motl RW. Accuracy and precision of smartphone applications and commercially available motion sensors in multiple sclerosis. *Multiple Sclerosis Journal - Experimental, Translational and Clinical* 2016;2((Balto J.M.; Kinnett-Hopkins D.L.; Motl R.W., robmotl@illinois.edu) Department of Kinesiology and Community Health, University of Illinois at Urbana-Champaign, United States) doi: 10.1177/2055217316634754
9. Beltrán-Carrillo VJ, Jiménez-Loaisa A, Alarcón-López M, et al. Validity of the "Samsung Health" application to measure steps: A study with two different samsung smartphones. *Journal of sports sciences* 2019;37(7):788-94. doi: 10.1080/02640414.2018.1527199
10. Block VJ, Zhao C, Hollenbach JA, et al. Validation of a consumer-grade activity monitor for continuous daily activity monitoring in individuals with multiple sclerosis. *Multiple Sclerosis Journal - Experimental, Translational and Clinical* 2019;5(4) doi: 10.1177/2055217319888660
11. Brodie MA, Pliner EM, Ho A, et al. Big data vs accurate data in health research: Large-scale physical activity monitoring, smartphones, wearable devices and risk of unconscious bias. *Medical Hypotheses* 2018;119((Gandevia S.C.; Lord S.R.) School of Medicine, University of New South Wales, Australia):32-36. doi: 10.1016/j.mehy.2018.07.015
12. Buckinx F, Mouton A, Reginster JY, et al. Relationship between ambulatory physical activity assessed by activity trackers and physical frailty among nursing home residents. *Gait and Posture* 2017;54((Appelboom G.) Byers Center for Biodesign, Stanford University, 318 Campus Drive E100, Stanford, CA, United States):56-61. doi: 10.1016/j.gaitpost.2017.02.010
13. Bunn JA, Jones C, Oliviera A, et al. Assessment of step accuracy using the Consumer Technology Association standard. *Journal of Sports Sciences* 2019;37(3):244-48. doi: 10.1080/02640414.2018.1491941
14. Burton E, Hill KD, Lautenschlager NT, et al. Reliability and validity of two fitness tracker devices in the laboratory and home environment for older community-dwelling people. *BMC geriatrics* 2018;18(1):103. doi: 10.1186/s12877-018-0793-4
15. Chandrasekar A, Hensor EMA, Mackie SL, et al. Preliminary concurrent validity of the Fitbit-Zip and ActiGraph activity monitors for measuring steps in people with polymyalgia rheumatica. *Gait and Posture* 2018;61((Harris E., E.Harris@hud.ac.uk) School of Human and Health Sciences, University of Huddersfield, Huddersfield, United Kingdom):339-45. doi: 10.1016/j.gaitpost.2018.01.035

16. Clay L, Webb M, Hargest C, et al. Gait quality and velocity influences activity tracker accuracy in individuals post-stroke. *Topics in Stroke Rehabilitation* 2019;26(6):412-17. doi: 10.1080/10749357.2019.1623474
17. De Ridder R, De Blaiser C. Activity trackers are not valid for step count registration when walking with crutches. *Gait and Posture* 2019;70((De Ridder R., Roel.DeRidder@ugent.be; De Blaiser C.) Department of Rehabilitation Sciences and Physiotherapy, Faculty of Medicine and Health Sciences, Ghent University, Ghent, Belgium):30-32. doi: 10.1016/j.gaitpost.2019.02.009
18. Duncan MJ, Wunderlich K, Zhao Y, et al. Walk this way: validity evidence of iphone health application step count in laboratory and free-living conditions. *Journal of sports sciences* 2018;36(15):1695-704. doi: 10.1080/02640414.2017.1409855
19. Ebara T, Azuma R, Shoji N, et al. Reliability of smartphone-based gait measurements for quantification of physical activity/inactivity levels. *Journal of occupational health* 2017;59(6):506-12. doi: 10.1539/joh.17-0101-OA
20. Floegel TA, Florez-Pregonero A, Hekler EB, et al. Validation of Consumer-Based Hip and Wrist Activity Monitors in Older Adults With Varied Ambulatory Abilities. *The journals of gerontology Series A, Biological sciences and medical sciences* 2017;72(2):229-36. doi: 10.1093/gerona/glw098
21. Fokkema T, Kooiman TJ, Krijnen WP, et al. Reliability and Validity of Ten Consumer Activity Trackers Depend on Walking Speed. *Medicine and science in sports and exercise* 2017;49(4):793-800. doi: 10.1249/MSS.0000000000001146
22. Gaz DV, Rieck TM, Peterson NW, et al. Determining the Validity and Accuracy of Multiple Activity-Tracking Devices in Controlled and Free-Walking Conditions. *American journal of health promotion : AJHP* 2018;32(8):1671-78. doi: 10.1177/0890117118763273
23. Hernández-Belmonte A, Bastida-Castillo A, Gómez-Carmona CD, et al. Validity and reliability of an inertial device (WIMU PROTM) to quantify physical activity level through steps measurement. *The Journal of sports medicine and physical fitness* 2019;59(4):587-92. doi: 10.23736/S0022-4707.18.08059-3
24. Höchsmann C, Knaier R, Eymann J, et al. Validity of activity trackers, smartphones, and phone applications to measure steps in various walking conditions. *Scandinavian journal of medicine & science in sports* 2018;28(7):1818-27. doi: 10.1111/sms.13074
25. Huang Y, Xu J, Yu B, et al. Validity of FitBit, Jawbone UP, Nike+ and other wearable devices for level and stair walking. *Gait and Posture* 2016;48((Huang Y.; Xu J.; Yu B.; Shull P.B., pshull@sjtu.edu.cn) State Key Laboratory of Mechanical System and Vibration, School of Mechanical Engineering, Shanghai Jiao Tong University, Shanghai, China):36-41. doi: 10.1016/j.gaitpost.2016.04.025
26. Hurt CP, Lein DH, Smith CR, et al. Assessing a novel way to measure step count while walking using a custom mobile phone application. *PLoS ONE* 2018;13(11) doi: 10.1371/journal.pone.0206828
27. Johnson M, Turek J, Dornfeld C, et al. Validity of the Samsung Phone S Health application for assessing steps and energy expenditure during walking and running: Does phone placement matter? *Digital health*;2:2055207616652747. doi: 10.1177/2055207616652747
28. Jones D, Crossley K, Dascombe B, et al. Validity and Reliability of the Fitbit Flex (Tm) and Actigraph Gt3x+ at Jogging and Running Speeds. *International Journal of Sports Physical Therapy* 2018;13(5):860-70. doi: 10.26603/ijspt20180860
29. Kendall B, Bellovary B, Gothe NP. Validity of wearable activity monitors for tracking steps and estimating energy expenditure during a graded maximal treadmill test. *Journal of sports sciences* 2019;37(1):42-49. doi: 10.1080/02640414.2018.1481723
30. Lamont RM, Daniel HL, Payne CL, et al. Accuracy of wearable physical activity trackers in people with Parkinson's disease. *Gait & posture* 2018;63:104-08. doi: 10.1016/j.gaitpost.2018.04.034

31. Lebleu J, Detrembleur C, Guebels C, et al. Concurrent validity of Nokia Go activity tracker in walking and free-living conditions. *Journal of Evaluation in Clinical Practice* 2020;26(1):223-28. doi: 10.1111/jep.13125
32. Leong JY, Wong JE. Accuracy of three Android-based pedometer applications in laboratory and free-living settings. *Journal of sports sciences* 2017;35(1):14-21.
33. Liew SJ, Gorny AW, Tan CS, et al. A Mobile Health Team Challenge to Promote Stepping and Stair Climbing Activities: Exploratory Feasibility Study. *JMIR mHealth and uHealth* 2020;8(2):e12665. doi: 10.2196/12665
34. Lu YT, Velipasalar S. Autonomous Footstep Counting and Traveled Distance Calculation by Mobile Devices Incorporating Camera and Accelerometer Data. *Ieee Sensors Journal* 2017;17(21):7157-66. doi: 10.1109/JSEN.2017.2752960
35. Magistro D, Brustio PR, Ivaldi M, et al. Validation of the ADAMO Care Watch for step counting in older adults. *PLoS ONE* 2018;13(2) doi: 10.1371/journal.pone.0190753
36. Major MJ, Alford M. Validity of the iPhone M7 motion co-processor as a pedometer for able-bodied ambulation. *Journal of sports sciences* 2016;34(23):2160-64.
37. Massouh F, Martin R, Chan B, et al. Is Activity Tracker-Measured Ambulation an Accurate and Reliable Determinant of Postoperative Quality of Recovery? A Prospective Cohort Validation Study. *Anesthesia and analgesia* 2019;129(4):1144-52. doi: 10.1213/ANE.0000000000003913
38. Montes J, Young JC, Tandy R, et al. Reliability and Validation of the Hexoskin Wearable Bio-Collection Device During Walking Conditions. *International journal of exercise science* 2018;11(7):806-16.
39. Montoye AHK, Mitrzyk JR, Molesky MJ. Comparative Accuracy of a Wrist-Worn Activity Tracker and a Smart Shirt for Physical Activity Assessment. *Measurement in Physical Education and Exercise Science* 2017;21(4):201-11. doi: 10.1080/1091367X.2017.1331166
40. Munck K, Christensen MH, Tahhan A, et al. Evaluation of Self-Trackers for Use in Telerehabilitation. *Journal of Usability Studies* 2018;13(3):125-37.
41. Orr K, Howe HS, Omran J, et al. Validity of smartphone pedometer applications. *BMC research notes* 2015;8((Faulkner G., guy.faulkner@ubc.ca) School of Kinesiology, University of British Columbia, Vancouver, Canada):733. doi: 10.1186/s13104-015-1705-8
42. Pepa L, Verdini F, Spalazzi L. Gait parameter and event estimation using smartphones. *Gait & posture* 2017;57:217-23. doi: 10.1016/j.gaitpost.2017.06.011
43. Polese JC, e Faria GS, Ribeiro-Samora GA, et al. Google fit smartphone application or Gt3X Actigraph: Which is better for detecting the stepping activity of individuals with stroke? A validity study. *Journal of Bodywork and Movement Therapies* 2019;23(3):461-65. doi: 10.1016/j.jbmt.2019.01.011
44. Presset B, Laurenczy B, Malatesta D, et al. Accuracy of a smartphone pedometer application according to different speeds and mobile phone locations in a laboratory context. *Journal of Exercise Science & Fitness* 2018;16(2):43-48. doi: 10.1016/j.jesf.2018.05.001
45. Psaltos D, Chappie K, Karahanoglu FI, et al. Multimodal Wearable Sensors to Measure Gait and Voice. *Digital Biomarkers* 2019;3(3):133-44. doi: 10.1159/000503282
46. Rüdiger S, Stuckenschneider T, Abeln V, et al. Validation of a widely used heart rate monitor to track steps in older adults. *The Journal of sports medicine and physical fitness* 2019;59(10):1622-27. doi: 10.23736/S0022-4707.19.09830-X
47. Schaffer SD, Holzapfel SD, Fulk G, et al. Step count accuracy and reliability of two activity tracking devices in people after stroke. *Physiotherapy theory and practice* 2017;33(10):788-96. doi: 10.1080/09593985.2017.1354412
48. Schmal H, Holsgaard-Larsen A, Izadpanah K, et al. Validation of Activity Tracking Procedures in Elderly Patients after Operative Treatment of Proximal Femur Fractures. *Rehabilitation Research and Practice* 2018;2018((Brønd J.C., jbrond@health.sdu.dk) Institute of Biomechanics and Sports Sciences, University of Southern Denmark, Denmark) doi: 10.1155/2018/3521271

49. Smith JD, Guerra G, Burkholder BG. The validity and accuracy of wrist-worn activity monitors in lower-limb prosthesis users. *Disability and rehabilitation* 2019((Guerra G.) Sirindhorn School of Prosthetics and Orthotics, Faculty of Medicine, Siriraj Hospital, Mahidol University, Bangkok, Thailand):1-7. doi: 10.1080/09638288.2019.1587792
50. Tam KM, Cheung SY. Validation of Electronic Activity Monitor Devices During Treadmill Walking. *Telemedicine journal and e-health : the official journal of the American Telemedicine Association* 2018;24(10):782-89. doi: 10.1089/tmj.2017.0263
51. Tedesco S, Sica M, Ancillao A, et al. Accuracy of consumer-level and research-grade activity trackers in ambulatory settings in older adults. *PLoS ONE* 2019;14(5) doi: 10.1371/journal.pone.0216891
52. Thorup CB, Andreasen JJ, Sørensen EE, et al. Accuracy of a step counter during treadmill and daily life walking by healthy adults and patients with cardiac disease. *BMJ Open* 2017;7(3) doi: 10.1136/bmjopen-2016-011742
53. Tophøj KH, Petersen MG, Sæbye C, et al. Validity and Reliability Evaluation of Four Commercial Activity Trackers' Step Counting Performance. *Telemedicine journal and e-health : the official journal of the American Telemedicine Association* 2018;24(9):669-77. doi: 10.1089/tmj.2017.0264
54. van Oeveren BT, de Ruiter CJ, Beek PJ, et al. An adaptive, real-time cadence algorithm for unconstrained sensor placement. *Medical Engineering & Physics* 2018;52:49-58. doi: 10.1016/j.medengphy.2017.12.007
55. Veerabhadrappa P, Moran MD, Renninger MD, et al. Tracking Steps on Apple Watch at Different Walking Speeds. *Journal of general internal medicine* 2018;33(6):795-96. doi: 10.1007/s11606-018-4332-y
56. Wahl Y, Düking P, Droszez A, et al. Criterion-validity of commercially available physical activity tracker to estimate step count, covered distance and energy expenditure during sports conditions. *Frontiers in Physiology* 2017;8(SEP) doi: 10.3389/fphys.2017.00725
57. Wong CK, Mentis HM, Kuber R. The bit doesn't fit: Evaluation of a commercial activity-tracker at slower walking speeds. *Gait & posture* 2018;59:177-81. doi: 10.1016/j.gaitpost.2017.10.010
58. Xie JQ, Wen D, Liang LZ, et al. Evaluating the Validity of Current Mainstream Wearable Devices in Fitness Tracking Under Various Physical Activities: Comparative Study. *Jmir Mhealth and Uhealth* 2018;6(4) doi: 10.2196/mhealth.9754
59. Bai Y, Hibbing P, Mantis C, et al. Comparative evaluation of heart rate-based monitors: Apple Watch vs Fitbit Charge HR. *Journal of sports sciences* 2018;36(15):1734-41. doi: 10.1080/02640414.2017.1412235
60. Bort-Roig J, Puig-Ribera A, Contreras RS, et al. Monitoring sedentary patterns in office employees: validity of an m-health tool (Walk@Work-App) for occupational health. *Gaceta sanitaria* 2018;32(6):563-66. doi: 10.1016/j.gaceta.2017.05.004
61. Genovese V, Mannini A, Sabatini AM. A Smartwatch Step Counter for Slow and Intermittent Ambulation. *Ieee Access* 2017;5:13028-37. doi: 10.1109/ACCESS.2017.2702066
62. Imboden MT, Nelson MB, Kaminsky LA, et al. Comparison of four Fitbit and Jawbone activity monitors with a research-grade ActiGraph accelerometer for estimating physical activity and energy expenditure. *British journal of sports medicine* 2018;52(13):844-50. doi: 10.1136/bjsports-2016-096990
63. Nelson MB, Kaminsky LA, Dickin DC, et al. Validity of Consumer-Based Physical Activity Monitors for Specific Activity Types. *Medicine and science in sports and exercise* 2016;48(8):1619-28. doi: 10.1249/MSS.0000000000000933
64. O'Connell S, G OL, Quinlan LR. When a Step Is Not a Step! Specificity Analysis of Five Physical Activity Monitors. *PLoS One* 2017;12(1):e0169616. doi: 10.1371/journal.pone.0169616 [published Online First: 2017/01/14]
65. Tedesco S, Sica M, Ancillao A, et al. Validity Evaluation of the Fitbit Charge2 and the Garmin vivosmart HR plus in Free-Living Environments in an Older Adult Cohort. *Jmir Mhealth and Uhealth* 2019;7(6) doi: 10.2196/13084

66. Ummels D, Beekman E, Theunissen K, et al. Counting Steps in Activities of Daily Living in People With a Chronic Disease Using Nine Commercially Available Fitness Trackers: Cross-Sectional Validity Study. *JMIR mHealth and uHealth* 2018;6(4):e70. doi: 10.2196/mhealth.8524
67. Wendel N, Macpherson CE, Webber K, et al. Accuracy of Activity Trackers in Parkinson Disease: Should We Prescribe Them? *Physical therapy* 2018;98(8):705-14. doi: 10.1093/ptj/pzy054
68. Amagasa S, Kamada M, Sasai H, et al. How Well iPhones Measure Steps in Free-Living Conditions: Cross-Sectional Validation Study. *JMIR mHealth and uHealth* 2019;7(1):e10418. doi: 10.2196/10418
69. Chu AHY, Ng SHX, Paknezhad M, et al. Comparison of wrist-worn Fitbit Flex and waist-worn ActiGraph for measuring steps in free-living adults. *Plos One* 2017;12(2) doi: 10.1371/journal.pone.0172535
70. Collins JE, Yang HY, Trentadue TP, et al. Validation of the Fitbit Charge 2 compared to the ActiGraph GT3X+ in older adults with knee osteoarthritis in free-living conditions. *PloS one* 2019;14(1):e0211231. doi: 10.1371/journal.pone.0211231
71. Degroote L, De Bourdeaudhuij I, Verloigne M, et al. The Accuracy of Smart Devices for Measuring Physical Activity in Daily Life: Validation Study. *JMIR mHealth and uHealth* 2018;6(12):e10972. doi: 10.2196/10972
72. Dominick GM, Winfree KN, Pohlig RT, et al. Physical Activity Assessment Between Consumer- and Research-Grade Accelerometers: A Comparative Study in Free-Living Conditions. *Jmir Mhealth and Uhealth* 2016;4(3) doi: 10.2196/mhealth.6281
73. Douma JAJ, Verheul HMW, Buffart LM. Feasibility, validity and reliability of objective smartphone measurements of physical activity and fitness in patients with cancer. *BMC Cancer* 2018;18(1) doi: 10.1186/s12885-018-4983-4
74. Ferguson T, Rowlands AV, Olds T, et al. The validity of consumer-level, activity monitors in healthy adults worn in free-living conditions: A cross-sectional study. *International Journal of Behavioral Nutrition and Physical Activity* 2015;12(1) doi: 10.1186/s12966-015-0201-9
75. Gill JMR, Hawari NSA, Maxwell DJ, et al. Validation of a Novel Device to Measure and Provide Feedback on Sedentary Behavior. *Medicine and science in sports and exercise* 2018;50(3):525-32. doi: 10.1249/MSS.0000000000001458
76. Gomersall SR, Ng N, Burton NW, et al. Estimating Physical Activity and Sedentary Behavior in a Free-Living Context: A Pragmatic Comparison of Consumer-Based Activity Trackers and ActiGraph Accelerometry. *Journal of medical Internet research* 2016;18(9):e239. doi: 10.2196/jmir.5531
77. Höchsmann C, Knaier R, Infanger D, et al. Validity of smartphones and activity trackers to measure steps in a free-living setting over three consecutive days. *Physiological measurement* 2020;41(1):015001. doi: 10.1088/1361-6579/ab635f
78. Hartwig TB, Del Pozo-Cruz B, White RL, et al. A monitoring system to provide feedback on student physical activity during physical education lessons. *Scandinavian journal of medicine & science in sports* 2019;29(9):1305-12. doi: 10.1111/sms.13438
79. Middelweerd A, H.P VDP, A VANH, et al. A Validation Study of the Fitbit One in Daily Life Using Different Time Intervals. *Medicine and science in sports and exercise* 2017;49(6):1270-79. doi: 10.1249/MSS.0000000000001225
80. Mooses K, Oja M, Reisberg S, et al. Validating Fitbit Zip for monitoring physical activity of children in school: a cross-sectional study. *BMC public health* 2018;18(1):858. doi: 10.1186/s12889-018-5752-7
81. Rosenberger ME, Buman MP, Haskell WL, et al. Twenty-four Hours of Sleep, Sedentary Behavior, and Physical Activity with Nine Wearable Devices. *Medicine and science in sports and exercise* 2016;48(3):457-65. doi: 10.1249/MSS.0000000000000778
82. Rozanski GM, Aqui A, Sivakumaran S, et al. Consumer Wearable Devices for Activity Monitoring Among Individuals After a Stroke: A Prospective Comparison. *JMIR cardio* 2018;2(1):e1. doi: 10.2196/cardio.8199

83. Stamatelopoulou A, Chapizanis D, Karakitsios S, et al. Assessing and enhancing the utility of low-cost activity and location sensors for exposure studies. *Environmental Monitoring and Assessment* 2018;190(3) doi: 10.1007/s10661-018-6537-2
84. Toth LP, Park S, Springer CM, et al. Video-Recorded Validation of Wearable Step Counters under Free-living Conditions. *Medicine and science in sports and exercise* 2018;50(6):1315-22. doi: 10.1249/MSS.0000000000001569
85. Voss C, Gardner RF, Dean PH, et al. Validity of Commercial Activity Trackers in Children With Congenital Heart Disease. *Canadian Journal of Cardiology* 2017;33(6):799-805. doi: 10.1016/j.cjca.2016.11.024
86. Yang X, Jago R, Zhang Q, et al. Validity and Reliability of the Wristband Activity Monitor in Free-living Children Aged 10-17 Years. *Biomedical and Environmental Sciences* 2019;32(11):812-22. doi: 10.3967/bes2019.103
87. O'Connell S, Ólaighin G, Quinlan LR. When a step is not a step! Specificity analysis of five physical activity monitors. *PLoS ONE* 2017;12(1) doi: 10.1371/journal.pone.0169616
